# Concurrent Diabetes Mellitus and Periodontitis in U.S. Adults: Mortality Associations and Machine Learning Risk Prediction

**DOI:** 10.3390/jcm15208138

**Published:** 2026-10-20

**Authors:** Jincheng Li, Tian Zhu, Haruhiko Kashiwazaki

**Affiliations:** Section of Geriatric Dentistry and Perioperative Medicine in Dentistry, Division of Maxillofacial Diagnostic and Surgical Science, Faculty of Dental Science, Kyushu University, Fukuoka 812-8582, Japan; li.jincheng.814@s.kyushu-u.ac.jp (J.L.); zhutian@dent.kyushu-u.ac.jp (T.Z.)

**Keywords:** diabetes mellitus, periodontitis, mortality, cardiovascular mortality, machine learning, inflammatory indices

## Abstract

**Background/Objectives**: We examined mortality associations by diabetes mellitus (DM)–periodontitis status and the incremental predictive value of composite inflammatory indices among adults with concurrent disease. **Methods**: We analyzed 10,632 adults from the National Health and Nutrition Examination Survey 2009–2014 linked to mortality data through 2019. Groups included neither DM nor periodontitis (NDM + NP), periodontitis alone (P), DM alone (DM), or concurrent DM and periodontitis (DM + P). Survey-weighted Cox and Fine–Gray models assessed all-cause and cardiovascular mortality. Among 978 participants with DM + P, nine survival models underwent five repeats of fivefold cross-validation to assess 5-year all-cause mortality prediction and the incremental value of eight composite inflammatory indices. **Results**: DM + P was associated with higher all-cause mortality than NDM + NP (HR, 1.51; 95% CI, 1.17–1.94) and P (HR, 1.31; 95% CI, 1.09–1.57). For cardiovascular mortality, DM + P was associated with higher mortality than NDM + NP (subdistribution hazard ratio [sHR], 1.51; 95% CI, 1.003–2.276), although the confidence interval only narrowly excluded 1.0. DM + P also had higher cardiovascular mortality than P (sHR, 1.55; 95% CI, 1.14–2.10). Neither outcome differed significantly between DM + P and DM. The Combined extreme gradient boosting Cox model had a concordance index and 5-year AUC of 0.754. Adding inflammatory indices did not significantly improve discrimination, prediction error, calibration, or net benefit. Observed 5-year mortality across predicted-risk tertiles was 3.7%, 7.4%, and 21.2%. **Conclusions**: Concurrent diabetes and periodontitis were associated with higher all-cause and cardiovascular mortality than periodontitis alone, but not with significantly higher mortality than diabetes alone. Composite inflammatory indices did not significantly improve prediction beyond clinical predictors.

## 1. Introduction

Periodontitis is characterized by inflammation and destruction of the tooth-supporting tissues, and severe periodontitis affected more than 1 billion people worldwide in 2021 [1,2]. Multiple studies have shown that diabetes mellitus (DM) is an established risk factor for periodontitis [3,4,5,6]. DM leads to exaggerated inflammatory responses to periodontal pathogens and also impairs resolution of inflammation and tissue repair, which leads to accelerated periodontal tissue destruction [7]. In addition, periodontitis patients had significantly higher prevalence of DM than participants with no periodontitis [8]. Periodontal treatment may improve glycemic control and reduce systemic inflammation in patients with DM and periodontitis [9,10,11,12]. These studies suggest a bidirectional relationship between DM and periodontitis.

DM is a significant risk factor for cardiovascular disease (CVD) and is associated with all-cause and cardiovascular mortality. Among DM patients, periodontitis was associated with a higher risk of developing diabetic complications compared to patients with no periodontitis [13]. A recent study using data from the National Health and Nutrition Examination Survey (NHANES) reported higher all-cause mortality in CVD patients with moderate or severe periodontitis than in those with no or mild periodontitis [14]. However, there is currently insufficient evidence to determine whether concurrent DM and periodontitis have a higher long-term mortality risk. Moreover, composite indices including the systemic immune-inflammation index, neutrophil-to-lymphocyte ratio, systemic inflammation response index, and prognostic nutritional index have been associated with mortality in diabetes and broader adult populations [15,16,17,18,19]. Whether these indices provide additional predictive value beyond clinical characteristics among adults with concurrent DM and periodontitis remains unclear.

We therefore conducted two analyses using NHANES data. First, we examined all-cause and cardiovascular mortality according to DM–periodontitis status and compared participants with both conditions with those who had either condition alone. Second, among participants with concurrent diabetes and periodontitis, we developed and internally validated time-to-event models. We assessed whether adding composite inflammatory indices provided incremental predictive value beyond clinical characteristics. Five-year prediction was prespecified as primary and 3-year prediction as secondary. We also explored whether adding the underlying raw laboratory components as separate predictors provided additional predictive information.

## 2. Materials and Methods

### 2.1. Study Design and Analytical Framework

We used data from the 2009–2010, 2011–2012, and 2013–2014 cycles of NHANES. NHANES uses a complex, multistage probability sampling design to represent the civilian, noninstitutionalized U.S. population. Standardized questionnaires, examinations, and laboratory procedures were used for data collection [20,21]. Interview, examination, and laboratory data were linked to the 2019 public-use linked mortality files, with follow-up through 31 December 2019.

The analysis comprised two linked components with different aims. The association component evaluated associations between DM–periodontitis status and mortality. The prediction component evaluated individual-level all-cause mortality prediction among participants with concurrent DM and periodontitis. NHANES protocols were approved by the National Center for Health Statistics Research Ethics Review Board. All participants provided written informed consent. The present analysis used publicly available, deidentified data.

### 2.2. Study Population

Participants were eligible for the association component if they were aged 30 years or older and had completed a periodontal examination. Eligibility also required sufficient information for DM and periodontitis classification, as well as available mortality follow-up and survey-design variables. Before complete-case restriction for Model 3 covariates, 11,734 participants met these criteria. A total of 1102 participants (9.4%) were excluded because of missing Model 3 covariates. The final analytic sample included 10,632 participants: 5282 with neither DM nor moderate-to-severe periodontitis (NDM + NP), 3475 with moderate-to-severe periodontitis alone (P), 897 with DM without moderate-to-severe periodontitis (DM), and 978 with concurrent DM and moderate-to-severe periodontitis (DM + P). Missing-data patterns and characteristics of included and excluded participants were examined in supplementary analyses (Supplementary Table S1).

The prediction component included all 978 participants with DM + P, among whom 202 all-cause deaths occurred. No formal sample-size calculation was performed because all eligible participants were included. Only 68 cardiovascular deaths occurred in this sample. We therefore did not develop a cardiovascular mortality prediction model because the event count was considered insufficient for model development, hyperparameter tuning, and internal evaluation.

### 2.3. Definitions of Diabetes and Periodontitis

DM was defined by a self-reported physician diagnosis, current insulin use, current use of oral glucose-lowering medication, or a measured hemoglobin A1c (HbA1c) level of 6.5% or higher [22]. Fasting plasma glucose was not included in the DM definition. 

Periodontitis was defined from full-mouth periodontal measurements using the Centers for Disease Control and Prevention/American Academy of Periodontology (CDC/AAP) surveillance criteria [23]. Third molars were excluded. Moderate periodontitis was defined as at least two interproximal sites with clinical attachment loss of 4 mm or greater on different teeth, or at least two interproximal sites with probing depth of 5 mm or greater on different teeth. Severe periodontitis was defined as at least two interproximal sites with clinical attachment loss of 6 mm or greater on different teeth and at least one interproximal site with probing depth of 5 mm or greater. Moderate and severe periodontitis were combined for the primary binary definition. The NP category included participants with no or mild periodontitis. Participants were classified as NDM + NP, P, DM, and DM + P. 

### 2.4. Mortality Outcomes and Covariates

Vital status and follow-up time were obtained from the NHANES public-use linked mortality files [24]. Follow-up extended from the baseline examination to death or administrative censoring on 31 December 2019. All-cause mortality included death from any cause. Cardiovascular mortality included diseases of heart and cerebrovascular diseases, corresponding to UCOD_LEADING recodes 001 and 005 [24]. Noncardiovascular death was treated as a competing event in the cardiovascular mortality analysis.

Covariates for the association analyses were selected before outcome modeling based on their epidemiological and clinical relevance to DM–periodontitis status and mortality, guided by established principles of confounder selection [25,26,27]. They included age, sex, race/ethnicity, educational attainment, poverty-to-income ratio, body mass index, smoking status, and hypertension. Race/ethnicity was categorized as Mexican American, other Hispanic, non-Hispanic White, non-Hispanic Black, or other race. Educational attainment was classified as less than college or college or above, and smoking as never or ever smoking. Hypertension was defined by a self-reported physician or health-professional diagnosis or current use of prescribed antihypertensive medication. Additional metabolic, lifestyle, healthcare-access, and disease-burden variables were evaluated in extended sensitivity analyses rather than included in the primary adjustment set, as some may reflect downstream consequences of DM or periodontitis [28].

### 2.5. Association Analyses

All association analyses accounted for the NHANES complex survey design using the combined 6-year examination weight, sampling strata, and primary sampling units. Continuous variables are presented as survey-weighted means with standard deviations, and categorical variables as unweighted numbers with survey-weighted percentages. Differences were assessed using design-adjusted Wald tests for continuous variables and Rao–Scott chi-square tests for categorical variables. 

Survey-weighted Kaplan–Meier curves and log-rank tests were used for all-cause survival. Survey-weighted Cox proportional hazards models estimated hazard ratios (HRs) and 95% confidence intervals (CIs) for all-cause mortality. Survey-weighted Aalen–Johansen curves described cardiovascular cumulative incidence, with noncardiovascular death treated as a competing event. Fine–Gray models estimated subdistribution hazard ratios (sHRs) and 95% CIs [29], and an unadjusted model provided the overall design-based Wald test. 

Three adjustment levels were prespecified. Model 1 was unadjusted. Model 2 included age, sex, race/ethnicity, and educational attainment. Model 3 additionally included poverty-to-income ratio, body mass index, smoking status, and hypertension. Each disease group was compared with NDM + NP. DM + P was also compared with P and DM.

Prespecified subgroup analyses were conducted by sex, age, race/ethnicity, smoking status, and body mass index. Interactions for DM + P versus P and DM + P versus DM were evaluated using fully adjusted survey-weighted Cox models with interaction terms. The multiplicative interaction between DM and periodontitis was evaluated using an interaction term between the two conditions. Subgroup and multiplicative-interaction analyses used survey-weighted Cox models. For cardiovascular mortality, noncardiovascular deaths were censored, and the resulting estimates were reported as cause-specific HRs. These cause-specific HRs were distinct from the Fine–Gray sHRs used in the primary cardiovascular mortality analysis (Supplementary Methods S1).

Missing-data sensitivity analyses compared participants included in and excluded from the complete-case analysis. Multiple imputation was also performed among the 11,734 eligible participants. The primary association models used the complete-case sample, with multiple imputation used to assess the robustness of the findings to missing covariate data (Supplementary Methods S2). In the prediction analyses, missing predictors were imputed within the training data as part of model preprocessing.

Additional sensitivity analyses included landmark analyses, diabetes-related characteristics, periodontitis severity, and broader covariate adjustment. Landmark analyses excluded deaths during the first 1 or 2 years and used survey-weighted Cox models. For cardiovascular mortality, noncardiovascular deaths were censored (Supplementary Methods S1). Diabetes-specific sensitivity analyses compared DM + P with DM in two subsets, with Model 3 first refitted separately within each subset. In the HbA1c-complete subset (n = 1830), HbA1c was added as the only additional covariate beyond Model 3. In the subset of participants with physician-diagnosed diabetes and complete data on diabetes-related variables (n = 1396), HbA1c, diabetes duration, current insulin use, and current use of diabetes pills were added together to Model 3. Moderate and severe periodontitis were also examined separately. For extended-covariate analyses, Model 3 was refitted in a common complete-case subset of 9358 participants. Extended-1 additionally adjusted for total cholesterol, alcohol exposure, physical activity, usual source of care, and health insurance status [30,31]. Extended-2 further adjusted for established cardiovascular disease, reduced kidney function, and albuminuria [32,33]. Detailed definitions and procedures are provided in Supplementary Methods S3 and S4.

The proportional hazards assumption for DM–periodontitis status in the Cox models and the proportional subdistribution hazards assumption for DM–periodontitis status in the Fine–Gray models were evaluated using time-interaction tests. All tests were two-sided, and no formal adjustment was made for multiple comparisons. 

### 2.6. Predictors for Mortality Prediction

Candidate predictors were selected a priori based on clinical relevance, baseline availability, and routine measurability. No predictors were selected based on univariable outcome associations [34]. The Clinical set included age, sex, race/ethnicity, educational attainment, smoking status, body mass index, HbA1c, hypertension, insulin use, and periodontitis severity (severe versus moderate).

The Inflammation set comprised the neutrophil-to-lymphocyte ratio (NLR), platelet-to-lymphocyte ratio (PLR), monocyte-to-lymphocyte ratio (MLR), systemic immune-inflammation index (SII), systemic inflammation response index (SIRI), aggregate index of systemic inflammation (AISI), platelet-to-high-density lipoprotein (HDL) cholesterol ratio (PHR), and prognostic nutritional index (PNI) [35]. These indices encompass inflammatory, nutritional, and platelet-to-lipid measures. The Combined set included both the Clinical and Inflammation predictors. Six underlying laboratory components were also evaluated in an exploratory analysis. These included neutrophil, lymphocyte, monocyte, and platelet counts, serum albumin, and HDL cholesterol. These variables were referred to as raw components. Definitions, transformations, and data-quality rules for the inflammatory indices and raw components are provided in Supplementary Methods S5.

### 2.7. Model Development and Internal Validation

The primary prediction analysis compared the Clinical and Combined extreme gradient boosting (XGBoost) Cox models. Across the three predictor sets and three modeling methods, nine candidate models were prespecified. Five-year performance was prespecified as primary, and 3-year performance as secondary. Harrell’s concordance index (C-index) was used to summarize discrimination over the available follow-up [36]. The three modeling methods were elastic-net Cox regression, random survival forest [37], and XGBoost Cox [38]. A conventional Clinical Cox model was used as a benchmark. A partially penalized Combined Cox model was evaluated as a sensitivity analysis.

The exploratory feature-representation analysis used the same XGBoost Cox procedure. Four models were compared: Clinical; Clinical + raw components; Clinical + composite indices; and Clinical + raw + composite. Shapley additive explanations (SHAP) interpretation and risk stratification were also exploratory.

Internal validation used five repeats of fivefold outer cross-validation, yielding 25 outer-fold evaluations. Outer and inner folds were stratified by death status and follow-up-time categories. The same outer folds were used for all models. Hyperparameters were selected within each outer training set. Each participant contributed one out-of-fold prediction per repeat, and the five predictions were averaged for performance evaluation.

All preprocessing was estimated from the relevant training data. Missing continuous and categorical predictors were imputed using training-set medians and modes, respectively (Supplementary Table S2). Absolute-risk estimation used the corresponding outer training set. Primary prediction analyses were unweighted because the target was individual-level performance within the observed DM + P sample. Examination-weighted performance was evaluated in a sensitivity analysis. Full implementation and tuning details are provided in the Supplementary Methods S6–S8.

### 2.8. Model Performance, Interpretation, and Risk Stratification

Performance was evaluated using Harrell’s C-index, the area under the time-dependent receiver operating characteristic curve (AUC), and Brier score [39,40,41]. AUC and Brier score were calculated at 3 and 5 years using inverse-probability-of-censoring weighting. Calibration was assessed using calibration plots, intercepts, and slopes [42]. Decision-curve net benefit was used to evaluate the potential clinical utility of the predictions [43]. Deaths and censoring before 3 and 5 years were summarized to assess the prespecified prediction horizons.

Uncertainty was estimated using 1000 paired participant-level bootstrap samples of the final out-of-fold prediction dataset. Predictions from competing models were resampled jointly, and the models were not refitted. The resulting CIs therefore reflected uncertainty conditional on the generated out-of-fold predictions and did not incorporate the full uncertainty from model development, preprocessing, and hyperparameter tuning. Percentile 95% CIs were calculated for model-specific performance estimates and paired differences. Two-sided bootstrap P values were calculated for paired differences. CIs for decision-curve analysis were pointwise and were not adjusted for multiple risk thresholds. CIs from the exploratory feature-representation analysis were also not adjusted for multiple comparisons.

Calibration slopes for the elastic-net Cox models and the conventional Clinical Cox benchmark were also checked across individual cross-validation repeats and alternative prediction-aggregation methods (Supplementary Methods S9).

SHAP values were calculated from an exploratory Combined XGBoost Cox model fitted to the full DM + P sample [44]. SHAP values represented contributions to the predicted log-relative hazard. They were not interpreted as causal effects or as changes in absolute 5-year mortality risk. Bootstrap procedures for SHAP uncertainty are described in the Supplementary Methods S10.

For descriptive risk stratification, participants were divided into equal-frequency tertiles of participant-averaged out-of-fold 5-year risk. Kaplan–Meier estimates and an unweighted log-rank test were used to compare the strata. Unadjusted unweighted Cox models were also fitted using the lowest predicted-risk tertile as the reference. The tertile cut points were derived from the present sample. The risk strata were descriptive and were not intended as clinical treatment thresholds. 

Analyses were performed in R version 4.5.3. Reporting of the association and prediction analyses was guided by the Strengthening the Reporting of Observational Studies in Epidemiology (STROBE) statement [45] and the Transparent Reporting of a multivariable prediction model for Individual Prognosis Or Diagnosis–Artificial Intelligence (TRIPOD+AI) statement [46], respectively.

## 3. Results

### 3.1. Study Population and Baseline Characteristics

**Figure 1:**
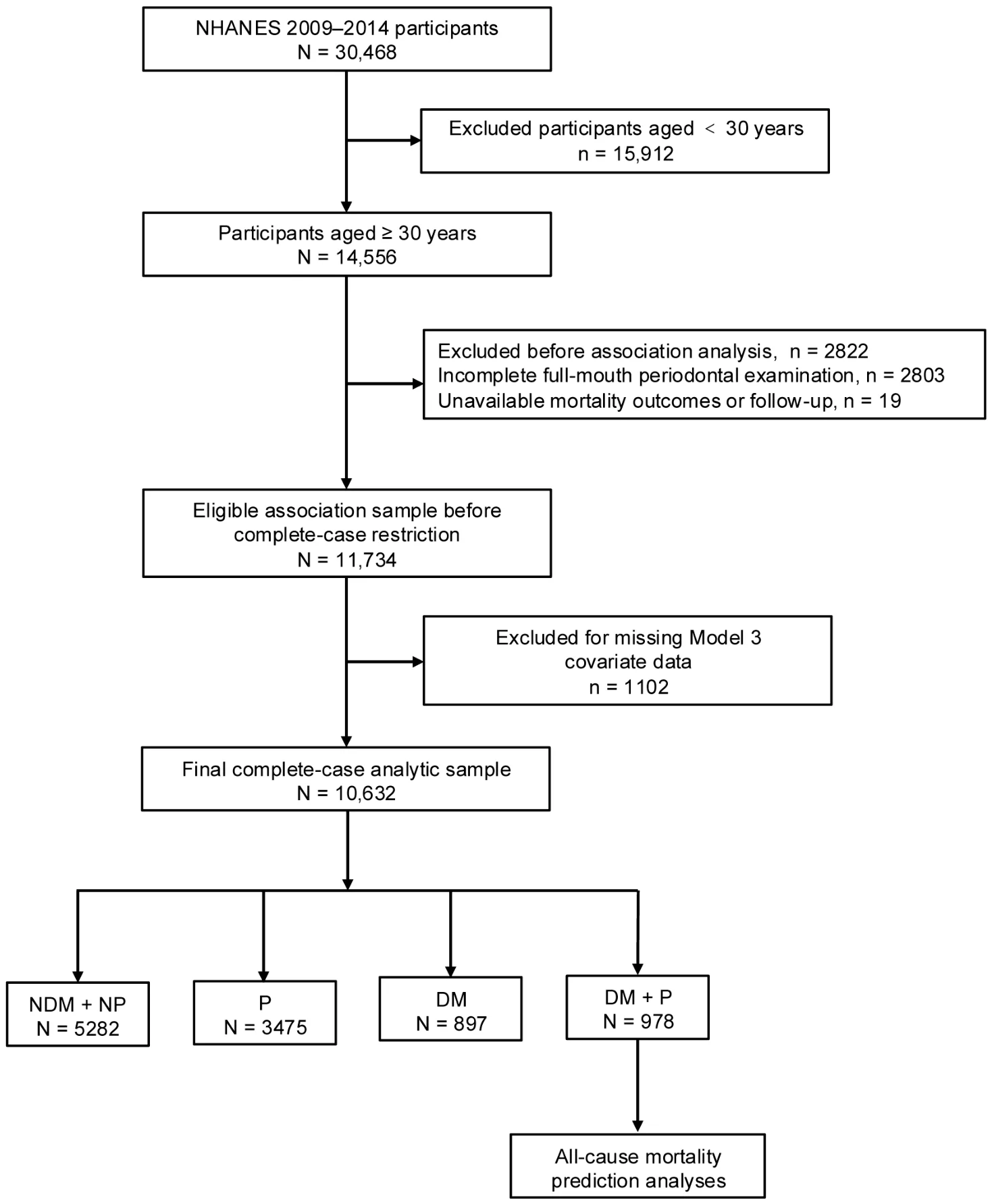
Study population selection. The flow diagram shows the selection of adults aged 30 years or older from the National Health and Nutrition Examination Survey (NHANES) 2009–2014. The final association sample included 10,632 participants, and the prediction analysis included the 978 participants with DM + P. NDM + NP, neither diabetes mellitus nor moderate-to-severe periodontitis; P, moderate-to-severe periodontitis alone; DM, diabetes mellitus without moderate-to-severe periodontitis; DM + P, concurrent diabetes mellitus and moderate-to-severe periodontitis.

The analytic sample included 10,632 participants: 5282 with NDM + NP, 3475 with P, 897 with DM, and 978 with DM + P (Figure 1). The mean age was 51.8 years, and 51.1% were women. Participants with DM or DM + P were older and had higher body mass index, HbA1c, and prevalence of hypertension. Ever smoking was more common among participants with P or DM + P. Men accounted for a higher proportion of participants in P and DM + P than in NDM + NP and DM. Baseline characteristics according to DM–periodontitis status are summarized in Table 1.

Before applying the complete-case restriction, 11,734 participants were eligible for the association analysis, and 1102 (9.4%) were excluded because of missing Model 3 covariates. Most missingness was due to poverty-to-income ratio data. The distribution of DM–periodontitis status differed between included and excluded participants (*p* = 0.021). All-cause mortality occurred in 8.5% of included participants and 11.1% of excluded participants (*p* = 0.048). Cardiovascular mortality status did not differ between included and excluded participants (*p* = 0.449) (Supplementary Table S1).

### 3.2. Mortality Across DM–Periodontitis Groups

**Figure 2:**
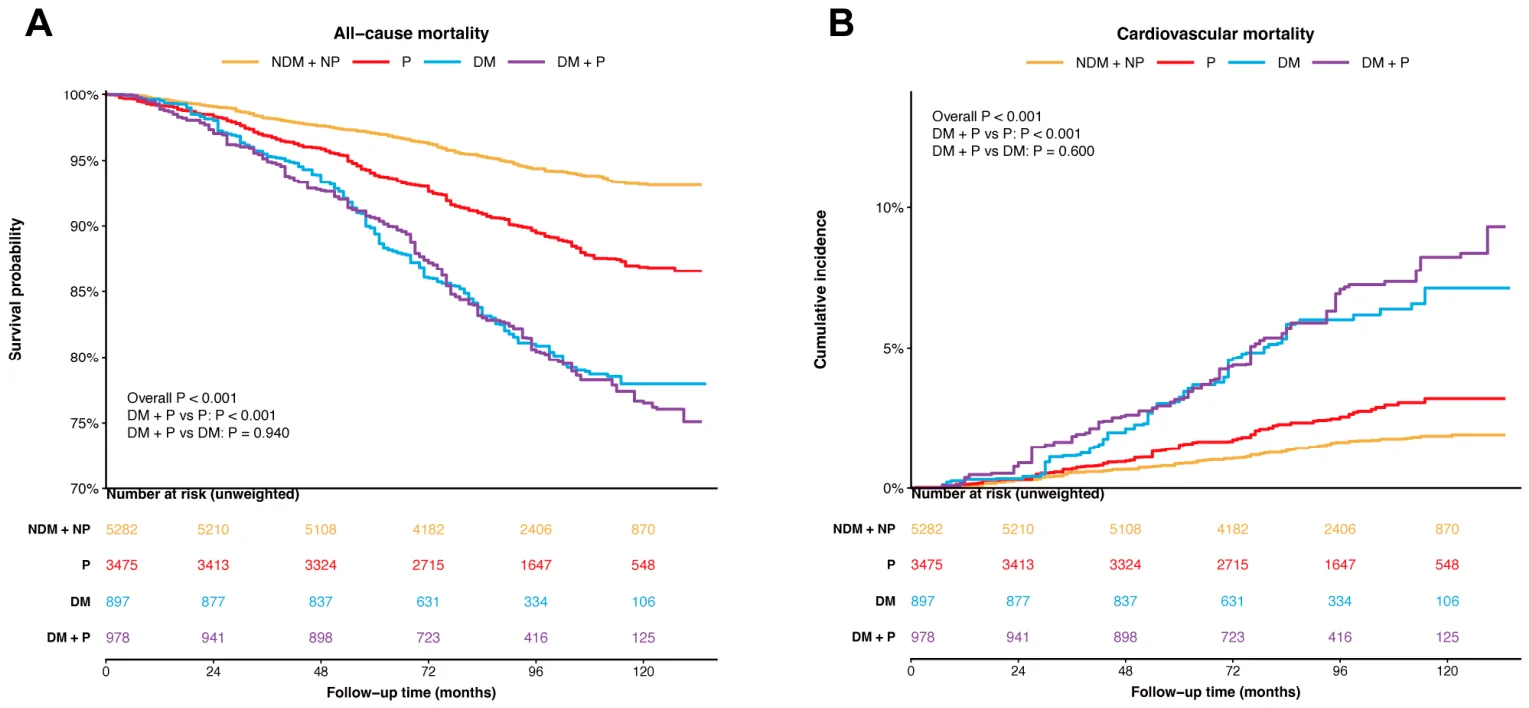
Survey-weighted all-cause survival and cardiovascular mortality according to DM–periodontitis status. (**A**) Survey-weighted Kaplan–Meier curves for all-cause survival. Overall and displayed pairwise *p* values were obtained from survey-weighted log-rank tests. (**B**) Survey-weighted Aalen–Johansen cumulative incidence curves for cardiovascular mortality. Overall and displayed pairwise *p* values were obtained from design-based Wald tests in unadjusted survey-weighted Fine–Gray models. Risk tables show unweighted numbers at risk. Group abbreviations are defined in Figure 1.

During a median follow-up of 7.67 years (interquartile range, 6.08–9.33), 1158 participants died, including 351 from cardiovascular causes. All-cause survival differed by DM–periodontitis status (survey-weighted log-rank *p* < 0.001). Survival was lower in DM + P than in P (*p* < 0.001). No statistically significant difference was observed between DM + P and DM (*p* = 0.940). Cardiovascular cumulative incidence also differed by DM–periodontitis status (survey-weighted Fine–Gray Wald *p* < 0.001), with pairwise *p* values of <0.001 for DM + P versus P and 0.600 for DM + P versus DM (Figure 2).

In the fully adjusted analyses, mortality associations differed across DM–periodontitis groups. For all-cause mortality, compared with NDM + NP, the HRs were 1.15 (95% CI, 0.95–1.40) for P, 1.51 (95% CI, 1.17–1.95) for DM, and 1.51 (95% CI, 1.17–1.94) for DM + P. DM + P was associated with higher all-cause mortality than P (HR, 1.31; 95% CI, 1.09–1.57; *p* = 0.003). For DM + P versus DM, the HR was 1.00 (95% CI, 0.73–1.35; *p* = 0.984) (Table 2).

For cardiovascular mortality, compared with NDM + NP, the fully adjusted sHRs were 0.98 (95% CI, 0.72–1.33) for P, 1.34 (95% CI, 0.95–1.90) for DM, and 1.51 (95% CI, 1.003–2.276; *p* = 0.048) for DM + P. DM + P was associated with higher cardiovascular mortality than P (sHR, 1.55; 95% CI, 1.14–2.10; *p* = 0.005). For DM + P versus DM, the sHR was 1.13 (95% CI, 0.71–1.79; *p* = 0.609) (Table 3).

### 3.3. Subgroup and Sensitivity Analyses

In exploratory subgroup analyses, the interaction *p* values for DM + P versus P were 0.030 for sex and 0.037 for age. The HRs were 1.73 (95% CI, 1.31–2.29) among women and 1.10 (95% CI, 0.85–1.42) among men. The corresponding HRs were 2.03 (95% CI, 1.34–3.09) among participants younger than 65 years and 1.14 (95% CI, 0.89–1.45) among those aged 65 years or older. For cardiovascular mortality, the age interaction *p* value was 0.013. The cause-specific HRs were 4.15 (95% CI, 1.76–9.81) among participants younger than 65 years and 1.16 (95% CI, 0.81–1.67) among those aged 65 years or older. No statistically significant interactions were observed for DM + P versus DM or in the remaining subgroup analyses (Supplementary Tables S3–S6; Supplementary Figures S1–S4).

The multiplicative interaction between DM and periodontitis was not statistically significant for all-cause mortality (*p* for interaction = 0.378) or in the cause-specific analysis of cardiovascular mortality (*p* for interaction = 0.575) (Supplementary Table S7). The estimates were similar in the landmark analyses. For DM + P versus P, the HRs for all-cause mortality were 1.33 (95% CI, 1.09–1.63) after excluding deaths during the first year and 1.32 (95% CI, 1.07–1.62) after excluding deaths during the first 2 years. The corresponding cause-specific HRs for cardiovascular mortality were 1.57 (95% CI, 1.11–2.23) and 1.57 (95% CI, 1.10–2.25). Estimates for DM + P versus DM remained nonsignificant at both landmarks for both outcomes (Supplementary Tables S8 and S9).

Multiple-imputation analyses produced similar all-cause mortality estimates. The HRs for DM + P were 1.45 (95% CI, 1.13–1.85) versus NDM + NP, 1.27 (95% CI, 1.06–1.51) versus P, and 0.93 (95% CI, 0.69–1.26) versus DM. For cardiovascular mortality, the sHR for DM + P versus P was 1.49 (95% CI, 1.13–1.95). The estimate versus NDM + NP was 1.43 (95% CI, 0.97–2.10; *p* = 0.071), and the estimate versus DM was 0.98 (95% CI, 0.66–1.45) (Supplementary Table S10).

Additional adjustment for diabetes-related characteristics did not materially change the comparison between DM + P and DM. After adjustment for HbA1c, the HR was 1.04 (95% CI, 0.75–1.44) for all-cause mortality and the sHR was 1.19 (95% CI, 0.74–1.93) for cardiovascular mortality. Among participants with physician-diagnosed diabetes, further adjustment for HbA1c, diabetes duration, insulin use, and current use of diabetes pills gave an HR of 1.07 (95% CI, 0.76–1.49) and an sHR of 1.18 (95% CI, 0.67–2.06), respectively (Supplementary Table S11).

Separating moderate and severe periodontitis did not show a clear severity gradient. Comparisons between severe and moderate periodontitis were not statistically significant for either all-cause or cardiovascular mortality among participants with or without DM. Comparisons of DM with moderate or severe periodontitis versus DM without moderate-to-severe periodontitis were also not statistically significant (Supplementary Table S12).

The extended-covariate analyses used a common complete-case subset of 9358 participants. Extended-1 additionally adjusted for total cholesterol, alcohol exposure, physical activity, usual source of care, and health insurance status. For all-cause mortality, the HRs for DM + P versus NDM + NP and P were 1.45 (95% CI, 1.11–1.89) and 1.19 (95% CI, 0.97–1.46), respectively. The HR for DM + P versus DM was 1.04 (95% CI, 0.73–1.47). For cardiovascular mortality, the sHRs for DM + P versus NDM + NP and P were 1.53 (95% CI, 0.99–2.37) and 1.57 (95% CI, 1.06–2.32), respectively. The sHR for DM + P versus DM was 1.23 (95% CI, 0.72–2.09) (Supplementary Table S13). Extended-2 further adjusted for established cardiovascular disease, reduced kidney function, and albuminuria. For all-cause mortality, the HRs for DM + P versus NDM + NP and P were 1.26 (95% CI, 0.97–1.63) and 1.03 (95% CI, 0.82–1.28), respectively. The HR for DM + P versus DM was 1.14 (95% CI, 0.81–1.61). For cardiovascular mortality, the sHRs for DM + P versus NDM + NP and P were 1.29 (95% CI, 0.82–2.02) and 1.35 (95% CI, 0.92–1.98), respectively. The sHR for DM + P versus DM was 1.48 (95% CI, 0.85–2.56). Comparisons between DM + P and DM remained statistically nonsignificant in both extended models (Supplementary Table S13).

### 3.4. Model Performance and Incremental Predictive Value

**Figure 3:**
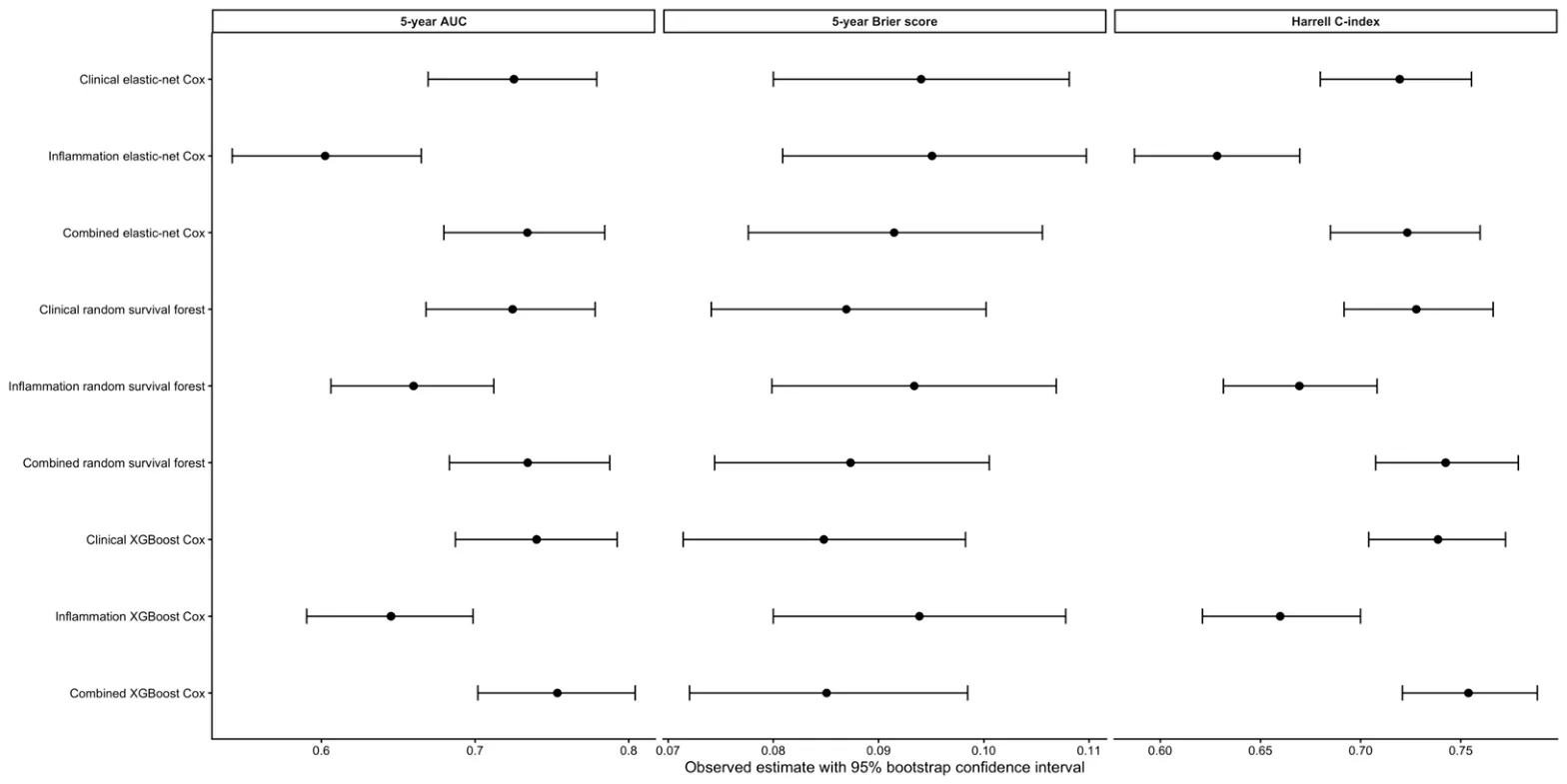
Internally validated performance of nine candidate time-to-event models in participants with DM + P. Points show performance estimates based on participant-level out-of-fold predictions, and horizontal bars show percentile 95% confidence intervals from 1000 participant-level bootstrap samples. Harrell’s concordance index (C-index) summarizes discrimination over the available follow-up. The area under the time-dependent receiver operating characteristic curve (AUC) and Brier score are shown at 5 years. Higher C-index and AUC values and lower Brier scores indicate better performance. XGBoost, extreme gradient boosting.

The prediction sample included all 978 participants with DM + P. During follow-up, 202 all-cause deaths occurred in this subgroup. At 5 years, 105 deaths had occurred, 2 participants (0.2%) had been censored before 5 years, and 871 participants remained under observation at the 5-year time point. In the primary comparison, adding the eight composite inflammatory indices to the Clinical XGBoost Cox model did not result in statistically significant differences in discrimination. The difference between the Combined and Clinical XGBoost Cox models was +0.015 (95% CI, −0.006 to 0.036) for the C-index and +0.014 (95% CI, −0.017 to 0.043) for the 5-year AUC. The 5-year Brier score, calibration, and decision-curve net benefit also did not favor the Combined model. Across the nine prespecified candidate models, the Combined XGBoost Cox model had a C-index of 0.754 (95% CI, 0.721–0.788), a 5-year AUC of 0.754 (95% CI, 0.702–0.804), and a 5-year Brier score of 0.0851 (95% CI, 0.0720–0.0985) (Figure 3; Supplementary Tables S14 and S15).

**Figure 4:**
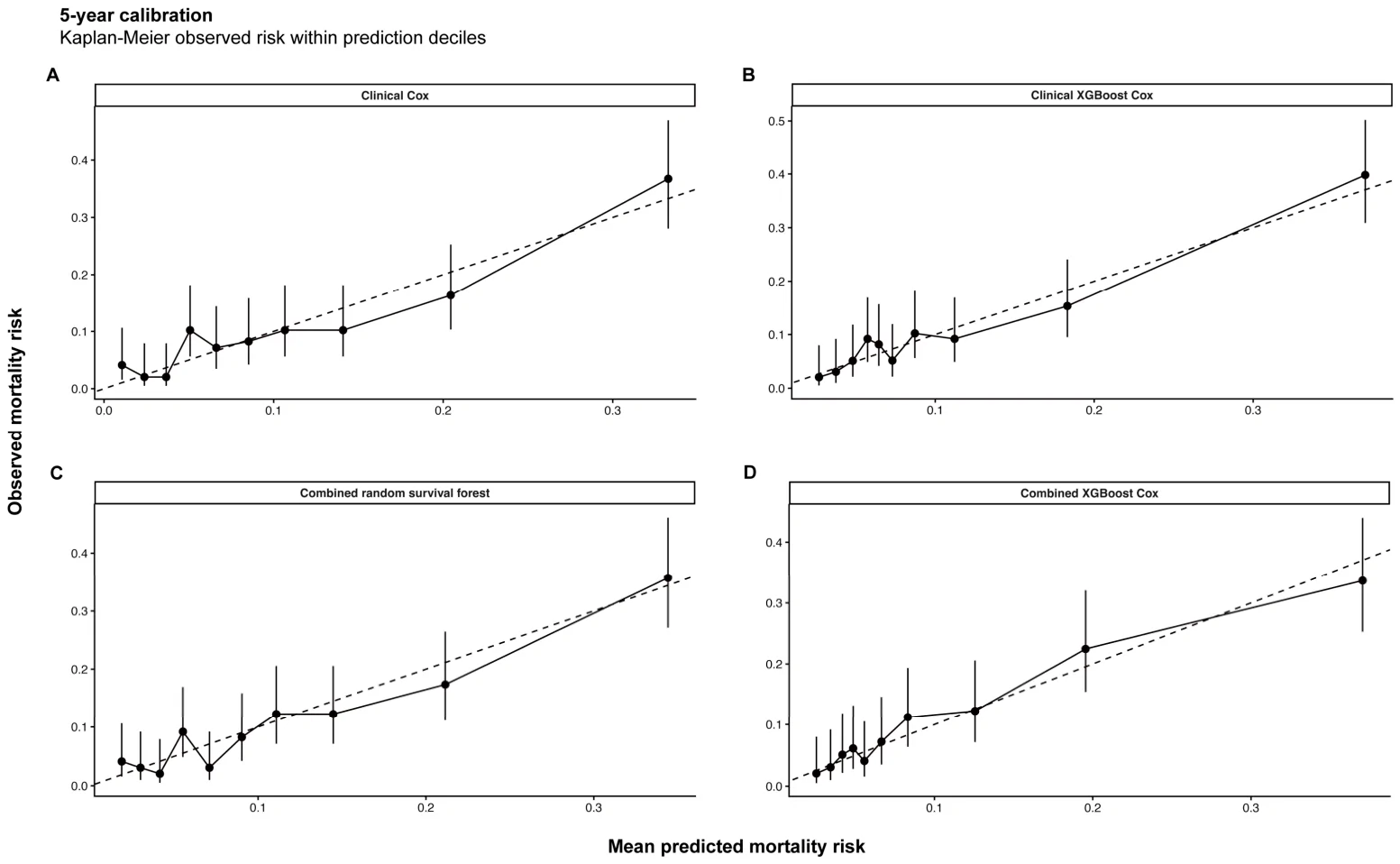
Five-year calibration of selected mortality-prediction models in participants with DM + P. (**A**) Clinical Cox; (**B**) Clinical XGBoost Cox; (**C**) Combined random survival forest; and (**D**) Combined XGBoost Cox. Points indicate Kaplan–Meier observed 5-year mortality within deciles of participant-averaged out-of-fold predicted risk, with vertical bars showing 95% confidence intervals. Solid lines connect the observed estimates, and the dashed diagonal line indicates perfect calibration. Calibration intercepts and slopes were estimated separately using inverse-probability-of-censoring-weighted complementary log–log regression.

Figure 4 shows 5-year calibration for four selected models. These models were chosen to include the conventional Clinical Cox benchmark, the Clinical and Combined XGBoost Cox models compared in the main prediction analysis, and the Combined random survival forest as a second machine learning approach. Panels A–D show the Clinical Cox, Clinical XGBoost Cox, Combined random survival forest, and Combined XGBoost Cox models, respectively. Across the four models, observed mortality generally increased with predicted risk, although the 95% CIs were wider at higher predicted risks. The Clinical and Combined XGBoost Cox models had calibration intercepts of 0.014 and 0.025 and slopes of 1.008 and 0.968, respectively. Calibration statistics for all nine models are provided in Supplementary Table S14. Additional checks reproduced the high calibration slopes observed for the elastic-net Cox models. For the Clinical elastic-net Cox model, the recalculated 5-year calibration slope was 6.34, identical to the stored estimate, and repeat-specific slopes ranged from 2.64 to 8.33. Alternative aggregation on the complementary log–log and cumulative-hazard scales yielded slopes of 6.45 and 6.31, respectively. The elastic-net Cox models also showed narrower distributions of predicted risks than the conventional Clinical Cox model (Supplementary Methods S9; Supplementary Table S16).

Decision-curve analysis did not identify an evaluated threshold at which the pointwise 95% CI favored the Combined over the Clinical XGBoost Cox model (Supplementary Figure S5). The Clinical Cox benchmark and the partially penalized Combined Cox model had similar discrimination and 5-year prediction error (Supplementary Table S17). The examination-weighted analysis also showed no statistically significant differences in C-index, 5-year AUC, or Brier score between the Clinical and Combined XGBoost Cox models (Supplementary Table S18).

In the exploratory feature-representation analysis, the Clinical + raw components XGBoost Cox model had a C-index of 0.759, a 5-year AUC of 0.764, and a 5-year Brier score of 0.0838 (Table 4). Compared with the Clinical model, the C-index was higher by 0.020 (95% CI, 0.001–0.041; *p* = 0.048). The 95% CIs for the differences in 5-year AUC and Brier score included zero. Adding the composite indices to the raw components produced little further change in these performance measures (Table 4). This exploratory analysis was not adjusted for multiple comparisons.

### 3.5. Risk Stratification and Model Interpretation

**Figure 5:**
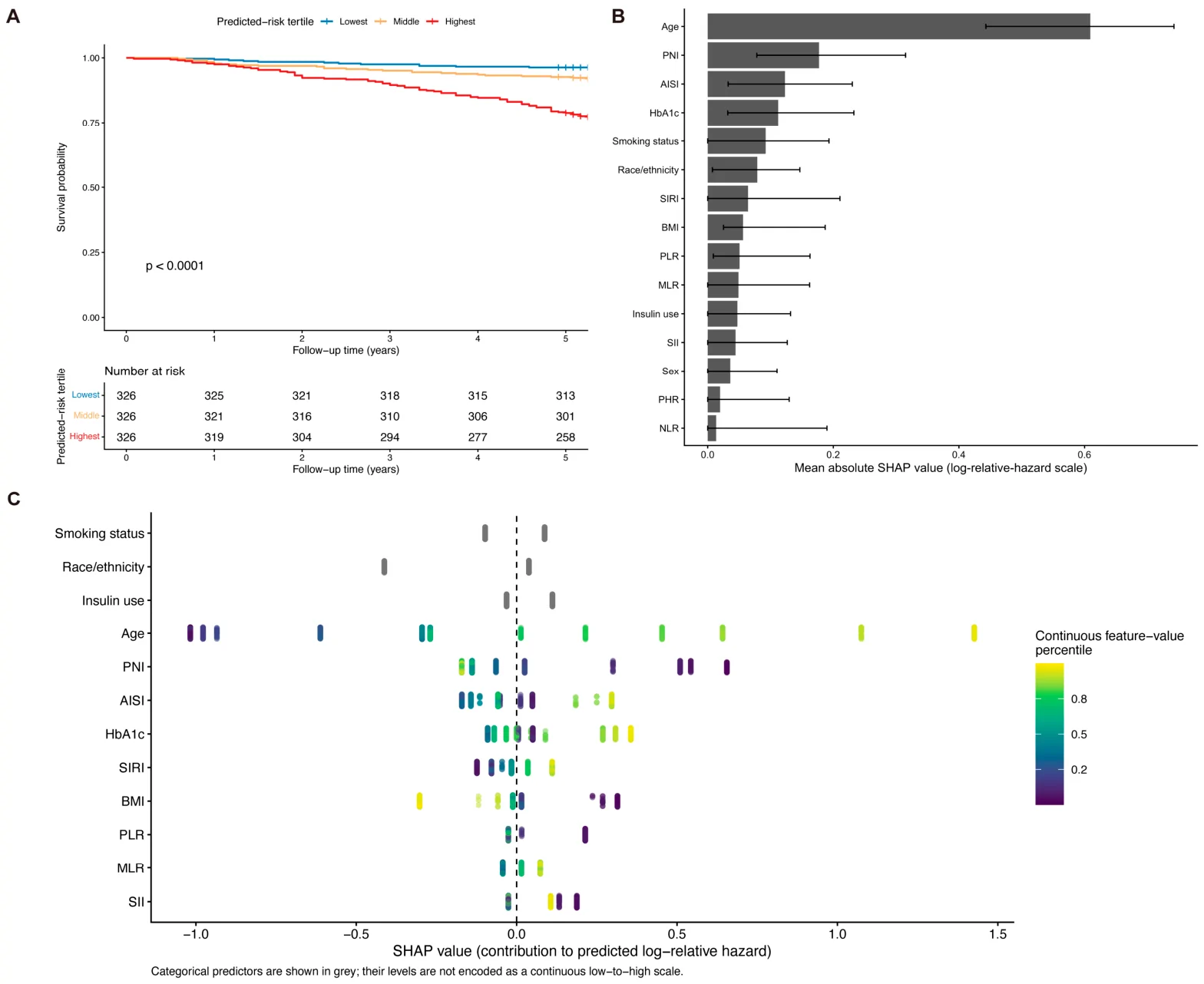
Risk stratification and model interpretation for the Combined XGBoost Cox model. (**A**) Kaplan–Meier curves according to equal-frequency tertiles of participant-averaged out-of-fold 5-year mortality risk. Tertile cut points were derived from the study sample and were descriptive rather than intended as clinical treatment thresholds. (**B**) Global feature importance based on mean absolute Shapley additive explanations (SHAP) values from the exploratory Combined XGBoost Cox interpretation model. Horizontal bars represent bootstrap 95% confidence intervals. (**C**) SHAP beeswarm plot for the 12 predictors with the highest mean absolute SHAP values, showing the distribution and direction of feature contributions to the model-predicted log-relative hazard. Continuous predictors are colored according to feature-value percentile, whereas categorical predictors are shown in gray. SHAP analyses were exploratory and should not be interpreted as causal effects or as changes in absolute 5-year mortality risk.

Participants were divided into equal-frequency tertiles based on participant-averaged out-of-fold 5-year mortality risk from the Combined XGBoost Cox model, with 326 participants in each tertile. The tertile cut points were derived from this study sample. Observed 5-year all-cause mortality was 3.7% (95% CI, 2.1%–6.4%) in the lowest predicted-risk tertile, 7.4% (95% CI, 5.0%–10.8%) in the middle predicted-risk tertile, and 21.2% (95% CI, 17.1%–26.0%) in the highest predicted-risk tertile (Figure 5A). Survival differed across the three tertiles (log-rank *p* < 0.001). In unadjusted Cox analyses, using the lowest predicted-risk tertile as the reference, the HRs were 2.26 (95% CI, 1.33–3.83) for the middle tertile and 8.59 (95% CI, 5.37–13.73) for the highest tertile (Supplementary Table S19). These tertiles were descriptive and were not intended as clinical treatment thresholds.

In the exploratory Combined XGBoost Cox interpretation model, age had the highest mean absolute SHAP value, followed by PNI, AISI, HbA1c, smoking status, race/ethnicity, and SIRI (Figure 5B,C). SHAP values represented contributions to the model-predicted log-relative hazard. They should not be interpreted as causal effects or as changes in absolute 5-year mortality risk.

## 4. Discussion

In this integrated NHANES analysis, DM + P was associated with higher all-cause and cardiovascular mortality than P, whereas no statistically significant difference was observed compared with DM. Compared with NDM + NP, DM + P was associated with higher mortality in the primary analyses, although the 95% CI for cardiovascular mortality only narrowly excluded 1.0 and the association was attenuated in the multiple-imputation analysis. In the fully adjusted models, no statistically significant multiplicative interaction between DM and periodontitis was observed. The coexistence of these conditions should not be interpreted as evidence of a synergistic mortality effect. Among participants with DM + P, adding eight composite inflammatory indices to the Clinical XGBoost Cox model did not demonstrate a statistically significant improvement in predictive performance. In the exploratory analysis, the Clinical + raw components model had a higher C-index than the Clinical model, whereas the differences in 5-year AUC and Brier score were not statistically significant.

The comparison between DM + P and DM remained nonsignificant after additional adjustment for HbA1c. Among participants with physician-diagnosed diabetes, further adjustment for diabetes duration, current insulin use, and current use of diabetes pills yielded a similar result. Separating moderate and severe periodontitis also did not reveal a clear mortality gradient. However, previous studies have reported associations between periodontal or oral health status and mortality in people with impaired glucose metabolism. Li et al. reported that among adults with DM, those with moderate or severe periodontitis had higher all-cause and cardiovascular mortality than those with no or mild periodontitis [47]. Tan et al. divided adults with prediabetes into periodontitis and nonperiodontitis groups. The periodontitis group had higher all-cause mortality than the nonperiodontitis group (HR, 1.28; 95% CI, 1.04–1.57) [48]. Wang et al. also reported that greater tooth loss was associated with higher all-cause and cardiovascular mortality among adults with DM [49]. Our study differs from these reports in several aspects. We focused on NHANES 2009–2014, which included full-mouth periodontal examinations, and defined periodontitis using CDC/AAP criteria. We also evaluated NDM + NP, P, DM, and DM + P within the same analytic framework. This allowed us to examine mortality according to the coexistence of two closely related chronic conditions. The analysis accounted for the NHANES complex survey design and treated noncardiovascular death as a competing event for cardiovascular mortality. Differences in study populations, periodontal exposure definitions, covariate adjustment, and outcome modeling may partly explain the variation across studies. In addition, the substantial mortality risk associated with diabetes itself may have made an additional association of periodontitis more difficult to detect. Residual confounding cannot be excluded, and the lack of a statistically significant difference between DM + P and DM should not be interpreted as evidence of equivalent mortality risk.

Covariate selection required balancing control of potential confounding against the risk of overadjustment [25,28]. Model 3 included selected demographic, socioeconomic, behavioral, and clinical covariates, while additional factors were assessed in sensitivity analyses. The broader adjustment models required a smaller common complete-case subset (9358 versus 10,632 participants), potentially affecting precision and the composition of the analytic sample. In the common complete-case subset, the HRs and sHRs for DM + P versus NDM + NP and P were lower after additional adjustment for total cholesterol, lifestyle, and healthcare-access factors (Extended-1), and decreased further after adjustment for established cardiovascular disease, reduced kidney function, and albuminuria (Extended-2). These latter variables may reflect pre-existing disease burden or downstream consequences of DM or periodontitis. Body mass index and hypertension, although included in Model 3, could also be influenced by established diabetes. Because the temporal relationships between these covariates and DM–periodontitis status could not be established, the extended models should not be considered inherently preferable adjustment sets.

Previous studies have reported associations of SII and NLR with mortality in adults with diabetes or prediabetes [15,16,17]. PNI has also been associated with mortality in type 2 diabetes [18], and SIRI has been associated with mortality in broader adult populations [19]. In our study, however, adding eight composite inflammatory indices to the Clinical XGBoost Cox model did not provide clear incremental predictive benefit beyond the Clinical model. The 95% CIs for the differences in C-index and 5-year AUC included zero, with no meaningful improvement in the 5-year Brier score, calibration, or net benefit. Because the Clinical and Combined models were evaluated using the same repeated cross-validation framework, their performance could be compared directly within the internal-validation setting. One possible explanation is that the Clinical model already included predictors such as age, HbA1c, smoking, body mass index, hypertension, and insulin use. The composite indices may therefore have provided little additional information beyond these variables. An association with mortality does not necessarily translate into additional predictive value when a marker is added to an existing model. Discrimination and prediction error describe different aspects of model performance [39,41], while calibration and net benefit provide further information on the accuracy and potential clinical use of predicted risks [42,50,51].

The exploratory analysis of raw components showed a different pattern. The Clinical + raw components model had a higher C-index than the Clinical model, although the 95% CIs for the differences in 5-year AUC and Brier score included zero. One possible explanation is that individual laboratory measurements may retain predictive information that is attenuated when they are combined into predefined composite indices. The lack of further improvement after adding composite indices to the raw components further suggests that these indices provided limited additional information beyond the individual measurements. However, the raw-component analysis was exploratory and was not adjusted for multiple comparisons. Its potential incremental predictive value therefore requires confirmation in independent data. 

Within the prediction sample, observed 5-year mortality ranged from 3.7% in the lowest predicted-risk tertile to 21.2% in the highest predicted-risk tertile. These tertiles were descriptive and were not intended as clinical treatment thresholds. Exploratory SHAP analysis identified age and several inflammatory, nutritional, and metabolic variables among the main contributors to model predictions. SHAP values reflect the contribution of individual variables to model predictions rather than causal effects. Before clinical application, the Combined XGBoost Cox model would require external validation in the intended populations and settings, with assessment of transportability and recalibration where needed [52,53,54,55]. Future impact studies should evaluate whether use of the model improves clinical decision-making or patient outcomes.

Several limitations should be considered. DM, periodontal status, laboratory measurements, and covariates were assessed only at baseline, so changes during follow-up could not be captured. Diabetes type could not be classified consistently across the included NHANES cycles, so type-specific mortality associations could not be examined. Although additional diabetes-related variables were considered in sensitivity analyses, residual confounding from unmeasured or incompletely measured factors cannot be excluded. The observational association estimates should not be interpreted as causal effects. The primary association analyses were based on complete cases, and selection bias related to missing covariate data remains possible despite the multiple-imputation sensitivity analysis. The prediction sample included 978 participants and 202 all-cause deaths, while only 68 cardiovascular deaths were available. Therefore, cardiovascular mortality prediction was not performed. The prediction analyses were unweighted and were intended for model development rather than population-representative estimation. The prediction models underwent internal validation but were not externally validated. Their transportability to other populations and settings therefore remains uncertain. Bootstrap CIs were conditional on the generated out-of-fold predictions and did not incorporate the full uncertainty from model fitting, preprocessing, and hyperparameter tuning. The raw components, subgroup, interaction, risk-stratification, and SHAP analyses were exploratory.

## 5. Conclusions

DM + P was associated with higher all-cause and cardiovascular mortality than P, whereas no statistically significant difference was observed compared with DM. Compared with NDM + NP, DM + P was associated with higher mortality in the primary analyses, although the cardiovascular association was less robust. Among participants with DM + P, observed 5-year mortality differed across descriptive predicted-risk tertiles. Adding eight composite inflammatory indices to clinical predictors did not provide clear incremental predictive benefit. The 95% CIs for the differences in C-index and 5-year AUC included zero, and no clear improvement was observed in prediction error, calibration, or net benefit. In the exploratory analysis, the Clinical + raw components model had a higher C-index than the Clinical model, while the 95% CIs for the differences in 5-year AUC and Brier score included zero. These findings suggest that the composite inflammatory indices provided limited additional predictive information beyond the clinical predictors in this sample, whereas the potential value of individual laboratory components requires further validation.

## Figures and Tables

**Table 1:** Baseline characteristics according to DM–periodontitis status.

Characteristic	Overall (n = 10,632)	NDM + NP (n = 5282)	P (n = 3475)	DM (n = 897)	DM + P (n = 978)	*p* Value
Percentage of participants, unweighted (%)	100	49.7	32.7	8.4	9.2	
Age, years	51.8 (13.9)	48.7 (13.4)	54.4 (13.6)	59.0 (13.5)	60.1 (11.8)	<0.001
Sex, n (%)						<0.001
Female	5370 (51.1)	3030 (56.3)	1438 (41.8)	504 (55.6)	398 (39.8)	
Male	5262 (48.9)	2252 (43.7)	2037 (58.2)	393 (44.4)	580 (60.2)	
Race/ethnicity, n (%)						<0.001
Mexican American	1363 (7.3)	526 (5.4)	542 (10.3)	87 (5.6)	208 (13.3)	
Other Hispanic	985 (5.0)	476 (4.6)	317 (5.4)	97 (5.8)	95 (5.9)	
Non-Hispanic White	4833 (70.2)	2776 (75.9)	1376 (63.0)	397 (67.7)	284 (53.5)	
Non-Hispanic Black	2225 (10.5)	881 (7.9)	838 (13.6)	222 (13.6)	284 (16.9)	
Other race	1226 (7.0)	623 (6.3)	402 (7.7)	94 (7.3)	107 (10.4)	
Educational attainment, n (%)						<0.001
Less than college	7899 (67.3)	3488 (59.0)	2839 (78.0)	735 (77.7)	837 (83.8)	
College or above	2733 (32.7)	1794 (41.0)	636 (22.0)	162 (22.3)	141 (16.2)	
Poverty-to-income ratio	3.09 (1.64)	3.38 (1.60)	2.69 (1.62)	2.81 (1.59)	2.54 (1.56)	<0.001
Body mass index, kg/m^2^	29.2 (6.6)	28.6 (6.3)	28.7 (6.2)	33.6 (7.4)	32.9 (7.5)	<0.001
HbA1c, %	5.7 (1.0)	5.4 (0.4)	5.5 (0.4)	7.2 (1.6)	7.5 (1.7)	<0.001
Smoking status, n (%)						<0.001
Never smoker	5694 (54.2)	3203 (61.4)	1563 (42.8)	462 (52.2)	466 (42.8)	
Ever smoker	4938 (45.8)	2079 (38.6)	1912 (57.2)	435 (47.8)	512 (57.2)	
Hypertension, n (%)						<0.001
No	6422 (64.1)	3624 (70.8)	2151 (64.3)	318 (36.3)	329 (32.6)	
Yes	4210 (35.9)	1658 (29.2)	1324 (35.7)	579 (63.7)	649 (67.4)	

Values are survey-weighted means (SDs) for continuous variables and unweighted numbers (survey-weighted percentages) for categorical variables. Hemoglobin A1c (HbA1c) values were summarized among participants with available measurements. *p* values were calculated using design-adjusted Wald tests for continuous variables and Rao–Scott chi-square tests for categorical variables. NDM + NP, neither diabetes mellitus nor moderate-to-severe periodontitis; P, moderate-to-severe periodontitis alone; DM, diabetes mellitus without moderate-to-severe periodontitis; DM + P, concurrent diabetes mellitus and moderate-to-severe periodontitis.

**Table 2:** Survey-weighted Cox regression for all-cause mortality.

Group	Deaths/Participants	Model 1 HR (95% CI)	Model 1 *p* Value	Model 2 HR (95% CI)	Model 2 *p* Value	Model 3 HR (95% CI)	Model 3 *p* Value
NDM + NP	400/5282	1.00 (Reference)	—	1.00 (Reference)	—	1.00 (Reference)	—
P	376/3475	1.98 (1.62–2.42)	<0.001	1.21 (0.99–1.47)	0.059	1.15 (0.95–1.40)	0.149
DM	180/897	3.66 (2.87–4.66)	<0.001	1.67 (1.29–2.15)	<0.001	1.51 (1.17–1.95)	0.001
DM + P	202/978	3.71 (2.91–4.72)	<0.001	1.65 (1.28–2.13)	<0.001	1.51 (1.17–1.94)	0.001
P	376/3475	1.00 (Reference)	—	1.00 (Reference)	—	1.00 (Reference)	—
DM + P	202/978	1.87 (1.58–2.21)	<0.001	1.37 (1.14–1.64)	<0.001	1.31 (1.09–1.57)	0.003
DM	180/897	1.00 (Reference)	—	1.00 (Reference)	—	1.00 (Reference)	—
DM + P	202/978	1.01 (0.76–1.35)	0.931	0.99 (0.74–1.33)	0.956	1.00 (0.73–1.35)	0.984

Deaths and participants are unweighted counts. Hazard ratios (HRs) and 95% confidence intervals (CIs) were estimated using survey-weighted Cox proportional hazards models accounting for National Health and Nutrition Examination Survey (NHANES) examination weights, sampling strata, and primary sampling units. Model 1 was unadjusted. Model 2 adjusted for age, sex, race/ethnicity, and educational attainment. Model 3 additionally adjusted for poverty-to-income ratio, body mass index, smoking status, and hypertension. Group abbreviations are defined in Table 1.

**Table 3:** Survey-weighted Fine–Gray competing-risk regression for cardiovascular mortality.

Group	Deaths/Participants	Model 1 sHR (95% CI)	Model 1 *p* Value	Model 2 sHR (95% CI)	Model 2 *p* Value	Model 3 sHR (95% CI)	Model 3 *p* Value
NDM + NP	117/5282	1.00 (Reference)	—	1.00 (Reference)	—	1.00 (Reference)	—
P	105/3475	1.67 (1.23–2.26)	0.001	0.96 (0.70–1.32)	0.815	0.98 (0.72–1.33)	0.882
DM	61/897	3.90 (2.74–5.54)	<0.001	1.53 (1.07–2.20)	0.019	1.34 (0.95–1.90)	0.099
DM + P	68/978	4.35 (3.01–6.29)	<0.001	1.76 (1.23–2.52)	0.002	1.51 (1.003–2.276)	0.048
P	105/3475	1.00 (Reference)	—	1.00 (Reference)	—	1.00 (Reference)	—
DM + P	68/978	2.61 (1.96–3.48)	<0.001	1.82 (1.42–2.34)	<0.001	1.55 (1.14–2.10)	0.005
DM	61/897	1.00 (Reference)	—	1.00 (Reference)	—	1.00 (Reference)	—
DM + P	68/978	1.12 (0.74–1.68)	0.597	1.14 (0.75–1.75)	0.535	1.13 (0.71–1.79)	0.609

Cardiovascular death was the event of interest, and noncardiovascular death was treated as a competing event. Subdistribution hazard ratios (sHRs) and 95% CIs were estimated using survey-weighted Fine–Gray models accounting for the 6-year NHANES examination weights, sampling strata, and primary sampling units. Model 1 was unadjusted. Model 2 adjusted for age, sex, race/ethnicity, and educational attainment. Model 3 additionally adjusted for poverty-to-income ratio, body mass index, smoking status, and hypertension. Group abbreviations are defined in Table 1.

**Table 4:** Exploratory comparison of XGBoost Cox feature representations.

Metric	Clinical	Clinical + Raw Components	Clinical + Composite Indices	Clinical + Raw + Composite	Paired Difference * (95% CI); *p* Value
Harrell C-index	0.739 (0.704–0.772)	0.759 (0.726–0.793)	0.754 (0.721–0.788)	0.756 (0.722–0.791)	+0.0205 (0.0011 to 0.0410); 0.048
5-year AUC	0.740 (0.687–0.792)	0.764 (0.716–0.814)	0.754 (0.702–0.804)	0.756 (0.706–0.808)	+0.0241 (−0.0032 to 0.0548); 0.088
5-year Brier score	0.085 (0.071–0.098)	0.084 (0.071–0.097)	0.085 (0.072–0.098)	0.085 (0.072–0.099)	+0.0010 (−0.0018 to 0.0037); 0.470
5-year calibration intercept	0.014 (−0.182–0.197)	0.015 (−0.176–0.189)	0.025 (−0.171–0.199)	0.014 (−0.179–0.188)	−0.0008 (−0.0362 to 0.0376); 0.979
5-year calibration slope	1.008 (0.802–1.230)	1.008 (0.826–1.210)	0.968 (0.775–1.171)	0.972 (0.784–1.170)	−0.0003 (−0.0968 to 0.1016); 0.931

Performance was evaluated using participant-averaged out-of-fold predictions from repeated fivefold internal validation. Raw components and composite indices are defined in Section 2.6. * For the concordance index (C-index) and the area under the time-dependent receiver operating characteristic curve (AUC), paired differences were calculated as Clinical + raw components minus Clinical; for the Brier score, the difference was Clinical minus Clinical + raw components. Calibration differences compare the absolute distance from the ideal values of 0 for the intercept and 1 for the slope. Confidence intervals and two-sided *p* values were obtained from 1000 paired participant-level bootstrap resamples. This analysis was exploratory.

## Data Availability

The data analyzed in this study are publicly available from the National Center for Health Statistics through the NHANES website and the public-use linked mortality files.

## Supplementary Materials

The following supporting information can be downloaded at https://www.mdpi.com/article/10.3390/jcm15208138/s1, Methods S1: Association Analyses; Methods S2: Missing-Data and Multiple-Imputation Analyses; Methods S3: Diabetes-Specific Sensitivity Analyses; Methods S4: Periodontitis-Severity and Extended-Covariate Sensitivity Analyses; Methods S5: Inflammatory Indices and Raw Components; Methods S6: Internal Validation and Preprocessing; Methods S7: Model Tuning and Absolute-Risk Estimation; Methods S8: Performance Evaluation and Sensitivity Analyses; Methods S9: Calibration-Slope Verification; Methods S10: SHAP Interpretation and Risk Stratification; Table S1: Model 3 covariate missingness and characteristics of participants included in and excluded from the complete-case analysis; Table S2: Missing data among candidate predictors; Table S3: Subgroup analyses of all-cause mortality comparing DM + P with P; Table S4: Subgroup analyses of all-cause mortality comparing DM + P with DM; Table S5: Subgroup analyses of cardiovascular mortality comparing DM + P with P; Table S6: Subgroup analyses of cardiovascular mortality comparing DM + P with DM; Table S7: Multiplicative interaction between DM and periodontitis in relation to all-cause and cardiovascular mortality; Table S8: Landmark analyses of mortality by DM–periodontitis status; Table S9: Landmark analyses of mortality comparing DM + P with either condition alone; Table S10: Complete-case and multiple-imputation estimates for mortality associations according to DM–periodontitis status; Table S11: Diabetes-specific sensitivity analyses for mortality comparing DM + P with DM; Table S12: Periodontitis-severity sensitivity analysis for all-cause and cardiovascular mortality; Table S13: Extended-covariate sensitivity analyses for all-cause and cardiovascular mortality; Table S14: Internally validated performance of the nine prespecified candidate models; Table S15: Paired comparison of the Clinical and Combined XGBoost Cox models; Table S16: Verification of 5-year calibration slopes and prediction dispersion; Table S17: Internally validated performance of the conventional Clinical Cox benchmark and the partially penalized Combined Cox sensitivity model; Table S18: Unweighted and examination-weighted performance of the Clinical and Combined XGBoost Cox models; Table S19: Descriptive risk stratification based on out-of-fold 5-year mortality risk; Figure S1: Subgroup analyses of all-cause mortality comparing DM + P with P; Figure S2: Subgroup analyses of all-cause mortality comparing DM + P with DM; Figure S3: Subgroup analyses of cardiovascular mortality comparing DM + P with P; Figure S4: Subgroup analyses of cardiovascular mortality comparing DM + P with DM; Figure S5: Five-year decision-curve analysis comparing the Clinical and Combined XGBoost Cox models. References [56,57,58,59,60,61,62,63] are cited in the Supplementary Materials.

## Abbreviations

The following abbreviations are used in this manuscript:

AUC	Area under the time-dependent receiver operating characteristic curve
CI	Confidence interval
CVD	Cardiovascular disease
DM	Diabetes mellitus
DM + P	Concurrent diabetes mellitus and periodontitis
HR	Hazard ratio
NDM + NP	Neither diabetes mellitus nor moderate-to-severe periodontitis
NHANES	National Health and Nutrition Examination Survey
P	Moderate-to-severe periodontitis alone
SHAP	SHapley Additive exPlanations
sHR	Subdistribution hazard ratio
