# Supplementary material for: Concurrent Diabetes Mellitus and Periodontitis in U.S. Adults: Mortality Associations and Machine Learning Risk Prediction
Source: J Clin Med. 2026 Oct 20;15(20):8138. doi: 10.3390/jcm15208138 (PMC13705780; doi:10.3390/jcm15208138)
Supplement: Supplementary file 1 [file jcm-15-08138-s001.zip › Revised Supplementary Materials.pdf]

# Supplementary Materials

## 1. Supplementary Methods

***Methods S1. Association Analyses***

The combined 6-year NHANES examination weight was calculated as WTMEC2YR/3 for the three survey cycles. Survey-weighted analyses incorporated the original sampling strata and primary sampling units.

For cardiovascular mortality, the 6-year examination weight was supplied during Fine–Gray data expansion. The resulting interval-specific weight therefore already incorporated the examination weight. The examination weight was not applied again. The expanded data were analyzed using the original NHANES sampling strata and primary sampling units.

Subgroup and landmark analyses used survey-weighted Cox models. For cardiovascular mortality, noncardiovascular deaths were censored and cause-specific HRs were reported.

The multiplicative-interaction analysis included binary indicators for DM and periodontitis and their product term. Interaction P values were obtained using design-based Wald tests. Proportional hazards assumptions for DM–periodontitis status were evaluated using joint Wald tests of status-by-log-time interactions.

***Methods S2. Missing-Data and Multiple-Imputation Analyses***

Before complete-case restriction, 11,734 participants met the eligibility criteria for the association analysis. Missingness was summarized for each Model 3 covariate. Characteristics of included and excluded participants were compared using the same survey-weighted procedures as the baseline comparisons.

Multiple imputation by chained equations was performed for the 11,734 eligible participants [56]. Education, poverty-to-income ratio, body mass index, smoking status, and hypertension were imputed. Predictive mean matching was used for poverty-to-income ratio and body mass index. Logistic regression was used for education, smoking status, and hypertension. DM–periodontitis status, age, sex, race/ethnicity, follow-up time, mortality outcomes, examination weight, sampling strata, and primary sampling units were included as predictors but were not imputed. Twenty imputed datasets were generated, with 20 iterations for each imputation.

Survey-weighted Model 3 Cox and Fine–Gray models were fitted in each imputed dataset. Model estimates were pooled using Rubin's rules [57]. The same mortality definitions and survey-design specifications were used as in the primary analyses.

***Methods S3. Diabetes-Specific Sensitivity Analyses***

Diabetes-specific sensitivity analyses included participants in the DM and DM + P groups. The first analysis was restricted to participants with available HbA1c. Model 3 was first refitted in this subset and was then additionally adjusted for HbA1c.

A second analysis was restricted to participants with physician-diagnosed diabetes and complete data on HbA1c, diabetes duration, current insulin use, and current diabetes-pill use. Diabetes duration was calculated as current age minus age at diabetes diagnosis. Model 3 was first refitted in this subset. HbA1c, diabetes duration, current insulin use, and current diabetes-pill use were then added to the model.

Survey-weighted Cox models were used for all-cause mortality. Survey-weighted Fine–Gray models were used for cardiovascular mortality, with noncardiovascular death treated as a competing event.

***Methods S4. Periodontitis-Severity and Extended-Covariate Sensitivity Analyses***

For the periodontitis-severity analysis, moderate and severe periodontitis were retained as separate categories. Participants were classified into six groups: NDM + NP, moderate P, severe P, DM, DM + moderate P, and DM + severe P. Survey-weighted Model 3 Cox models were used for all-cause mortality. Survey-weighted Fine–Gray models were used for cardiovascular mortality.

Extended-covariate analyses used a common complete-case subset of 9,358 participants for all model comparisons. Model 3 was first refitted in this subset. Extended-1 additionally adjusted for total cholesterol, alcohol exposure, physical activity, usual source of care, and health insurance status. Alcohol exposure was defined as a history of consuming at least 12 alcoholic drinks in any one year. Physical activity was defined as any reported vigorous or moderate work activity, walking or bicycling for transportation, or vigorous or moderate recreational activity. Usual source of care was defined as reporting one or more places usually used for care when sick or needing health advice. Health insurance status was defined by self-reported coverage by health insurance or another health care plan.

Extended-2 further adjusted for established cardiovascular disease, reduced kidney function, and albuminuria. Established cardiovascular disease was defined by a self-reported history of congestive heart failure, coronary heart disease, angina, myocardial infarction, or stroke. Estimated glomerular filtration rate was calculated using the 2021 CKD-EPI creatinine equation [58]. Reduced kidney function was defined as an estimated glomerular filtration rate below 60 mL/min/1.73 $m^2$. Albuminuria was defined as a urinary albumin-to-creatinine ratio of 30 mg/g or higher [59].

### *Methods S5. Inflammatory Indices and Raw Components*

Absolute neutrophil, lymphocyte, and monocyte counts and platelet counts were measured in $10^3$ cells/μL. Serum albumin was measured in g/dL and HDL cholesterol in mg/dL. The composite inflammatory indices were calculated as follows: NLR = neutrophil/lymphocyte; PLR = platelet/lymphocyte; MLR = monocyte/lymphocyte; SII = platelet × neutrophil/lymphocyte; SIRI = neutrophil × monocyte/lymphocyte; AISI = platelet × neutrophil × monocyte/lymphocyte; PHR = platelet/HDL cholesterol; and PNI = 10 × albumin + 5 × lymphocyte count [60-63]. The six underlying laboratory variables—neutrophil count, lymphocyte count, monocyte count, platelet count, albumin, and HDL cholesterol—were evaluated as raw components.

Negative or nonfinite laboratory values were treated as missing. Zero values were treated as missing when a positive value was required or when the variable was used as a denominator. Nonfinite derived indices were also treated as missing. For linear Cox models, NLR, PLR, MLR, SII, SIRI, AISI, and PHR were transformed using log(1 + x), whereas PNI remained on its original scale. Tree-based models used the untransformed values.

The exploratory feature-representation analysis compared four XGBoost Cox models: Clinical, Clinical + raw components, Clinical + composite indices, and Clinical + raw + composite indices. The same resampling assignments were used for all four models.

### *Methods S6. Internal Validation and Preprocessing*

Internal validation used five repeats of fivefold outer cross-validation. This produced 25 held-out outer-fold evaluations. The same outer folds were used across predictor sets and algorithms.

Outer and inner folds were stratified by all-cause mortality status and follow-up-time quartiles where feasible. Hyperparameters for elastic-net Cox and XGBoost Cox models were selected using fivefold inner cross-validation within each outer training set. Random survival forest hyperparameters were selected using out-of-bag prediction error within each outer training set.

All preprocessing was estimated from the relevant training data. Continuous predictors were imputed using the training median. Categorical predictors were imputed using the training mode. Categorical levels were determined from the training data. Dummy encoding was used for penalized Cox and XGBoost models, whereas random survival forests used factor variables directly.

Penalized Cox models used training-data standardization during model fitting. The conventional Clinical Cox model was fitted on the imputed original predictor scale. Tree-based models were not scaled. Each participant received one out-of-fold prediction in each repeat. The five predictions were then averaged to obtain the participant-level out-of-fold prediction used for performance evaluation.

### *Methods S7. Model Tuning and Absolute-Risk Estimation*

Elastic-net Cox regression was evaluated using alpha values of 0.10, 0.25, 0.50, 0.75, and 1.00. Thirty lambda values were considered, with a minimum lambda ratio of 0.01. Mean inner-validation Harrell C-index was used for tuning.

The 1-standard-error rule was applied to select lambda within each alpha value. The final alpha–lambda combination was selected according to inner-validation C-index.

The partially penalized Combined Cox model used the same tuning procedure. Clinical predictors were not penalized. The eight composite inflammatory indices were penalized.

Random survival forests used log-rank splitting. Candidate terminal-node sizes were 5, 10, 15, 20, and 30. Node size and mtry were selected using out-of-bag prediction error from 250-tree tuning forests. The final models used 1,000 trees.

XGBoost Cox models used maximum tree depths of 1, 2, and 3. Learning rates of 0.03 and 0.08 and minimum child weights of 5 and 10 were evaluated. Subsample and column-subsample fractions were fixed at 0.80. Gamma and the L1 penalty were set to 0, and the L2 penalty was set to 1. Cox negative log-likelihood was minimized over a maximum of 400 boosting rounds. Early stopping was applied after 30 rounds without improvement.

For Cox-type models, absolute mortality risks were calculated using a Breslow baseline cumulative hazard estimated from the corresponding outer training set. For random survival forests, mortality risk was calculated as one minus the predicted survival probability at 3 or 5 years.

### *Methods S8. Performance Evaluation and Sensitivity Analyses*

Harrell C-index was calculated from participant-averaged out-of-fold risk scores. Time-dependent AUC and Brier score were evaluated at 3 and 5 years using inverse-probability-of-censoring weighting. Deaths and censoring before 3 and 5 years were summarized to assess the prespecified prediction horizons.

Five-year calibration intercepts and slopes were estimated using IPCW-weighted complementary log–log regression. Calibration plots used 10 equal-frequency groups of predicted risk. Kaplan–Meier estimates were used for observed mortality within each group.

Decision-curve analysis used participant-averaged out-of-fold 5-year risks. Censoring was handled using inverse-probability-of-censoring weighting, with the censoring distribution estimated using the Kaplan–Meier method. Threshold probabilities ranged from 0.01 to 0.30 in increments of 0.01. The Clinical and Combined XGBoost Cox models were compared with treat-all and treat-none strategies. Censoring weights and net benefits were recalculated within each paired participant-level bootstrap resample. Confidence intervals for net-benefit differences were pointwise and were not adjusted for multiple thresholds.

Confidence intervals and paired comparisons were obtained from 1,000 participant-level bootstrap resamples of the final out-of-fold prediction dataset. Predictions from competing models were resampled together. Models were not retrained during bootstrap resampling. These bootstrap intervals reflect uncertainty conditional on the final out-of-fold predictions. They do not capture the full uncertainty arising from model development, preprocessing, and hyperparameter tuning.

For C-index and AUC, paired differences were calculated as Combined minus Clinical. For Brier score, the difference was calculated as Clinical minus Combined. Calibration differences compared the absolute distance from the ideal value. The ideal value was 0 for the intercept and 1 for the slope.

The examination-weighted sensitivity analysis used normalized 6-year NHANES examination weights with a mean of 1. Weighted censoring estimation and weighted aggregation of performance measures were used. Sampling strata and primary sampling units were not included in variance estimation. Therefore, this analysis was not considered a full design-based prediction analysis.

### *Methods S9. Calibration-Slope Verification*

Five-year calibration slopes were recalculated for the elastic-net Cox models and the conventional Clinical Cox benchmark. The recalculation used the final participant-averaged out-of-fold predictions and the same IPCW-weighted complementary log–log regression as in the primary analysis.

Calibration slopes were also calculated separately within each of the five cross-validation repeats. Each repeat contained one out-of-fold prediction per participant. Two alternative approaches were used to combine repeated predictions. Repeat-specific predictions were averaged on the complementary log–log scale or on the cumulative-

hazard scale and then transformed back to absolute risk. These estimates were compared with those based on the prespecified arithmetic mean of absolute risks.

Prediction dispersion was compared across the aggregation methods and with the conventional Clinical Cox model. No model retraining or hyperparameter reselection was performed for these verification analyses.

***Methods S10. SHAP Interpretation and Risk Stratification***

The exploratory Combined XGBoost Cox interpretation model was fitted using all 978 participants with DM + P. Hyperparameters were selected using a separate fivefold cross-validation procedure. The selected model was then refitted using the full prediction sample. This model was used only for interpretation and was not used to estimate internally validated model performance.

SHAP values were calculated from the raw XGBoost Cox model output. They therefore represented additive contributions to the predicted log-relative hazard. Dummy-variable SHAP values for categorical predictors were combined at the original predictor level. Global feature importance was summarized using mean absolute SHAP values.

SHAP uncertainty was evaluated using 1,000 participant-level bootstrap samples. Preprocessing and model fitting were repeated within each bootstrap sample. The previously selected hyperparameters were kept fixed.

For descriptive risk stratification, participants were divided into equal-frequency tertiles of participant-averaged out-of-fold 5-year mortality risk from the Combined XGBoost Cox model. Observed 3- and 5-year mortality risks were estimated using Kaplan–Meier methods. Survival differences were evaluated using an unweighted log-rank test. Unadjusted unweighted Cox models used the lowest predicted-risk tertile as the reference. The tertile cut points were derived from this study sample. The tertiles were descriptive and were not selected to optimize outcome separation or define clinical treatment thresholds.

## 2. Supplementary Tables

**Supplementary Table S1.** Model 3 covariate missingness and characteristics of participants included in and excluded from the complete-case analysis.

| Characteristic | Missing among 11,734 eligible participants, n (%) | Included complete-case sample (n = 10,632) | Excluded for missing Model 3 covariates (n = 1,102) | P value |
|---|---|---|---|---|
| **Model 3 covariate missingness** | | | | |
| Age | 0 (0.00) | — | — | — |
| Sex | 0 (0.00) | — | — | — |
| Race/ethnicity | 0 (0.00) | — | — | — |
| Educational attainment | 15 (0.13) | — | — | — |
| Poverty-to-income ratio | 997 (8.50) | — | — | — |
| Body mass index | 91 (0.78) | — | — | — |
| Smoking status | 4 (0.03) | — | — | — |
| Hypertension | 16 (0.14) | — | — | — |
| Any Model 3 covariate missing | 1102 (9.39) | — | — | — |
| **Characteristics of included and excluded participants** | | | | |
| Participants, unweighted n | — | 10,632 | 1,102 | — |
| Age, years | — | 51.78 (13.92) | 53.00 (14.53) | 0.114 |
| Follow-up, years | — | 7.64 (2.02) | 7.59 (2.27) | 0.703 |
| Sex, survey-weighted % | | | | — |
| Female | — | 51.1 | 54.2 | |
| Male | — | 48.9 | 45.8 | |
| Race/ethnicity, survey-weighted % | | | | — |
| Mexican American | — | 7.3 | 12.9 | |
| Other Hispanic | — | 5.0 | 10.0 | |
| Non-Hispanic White | — | 70.2 | 52.4 | |
| Non-Hispanic Black | — | 10.5 | 13.8 | |
| Other race | — | 7.0 | 10.9 | |
| DM–periodontitis status, survey-weighted % | | | | 0.021 |
| NDM + NP | — | 58.0 | 51.7 | |
| P | — | 28.5 | 34.3 | |
| DM | — | 7.1 | 7.4 | |
| DM + P | — | 6.4 | 6.6 | |
| All-cause mortality status, survey-weighted % | | | | 0.048 |
| Alive | — | 91.5 | 88.9 | |
| Death | — | 8.5 | 11.1 | |
| Cardiovascular mortality status, survey-weighted % | | | | 0.449 |
| No cardiovascular death | — | 97.6 | 97.2 | |
| Cardiovascular death | — | 2.4 | 2.8 | |

Missingness is presented as unweighted n (%). Characteristics are presented as survey-weighted means (SDs) for continuous variables and survey-weighted percentages for categorical variables; participant counts are unweighted. Missingness counts for individual Model 3 covariates are not mutually exclusive. P values were calculated using design-adjusted Wald tests for continuous variables and Rao–Scott chi-square tests for categorical variables, where available. NDM + NP, neither diabetes mellitus nor moderate-to-severe periodontitis; P, moderate-to-severe periodontitis alone; DM, diabetes mellitus without moderate-to-severe periodontitis; DM + P, concurrent diabetes mellitus and moderate-to-severe periodontitis.

**Supplementary Table S2.** Missing data among candidate predictors.

| Predictor | Missing, n (%) |
|---|---|
| HbA1c | 25 (2.56) |
| NLR | 24 (2.45) |
| PLR | 24 (2.45) |
| MLR | 24 (2.45) |
| SII | 24 (2.45) |
| SIRI | 24 (2.45) |
| AISI | 24 (2.45) |
| PHR | 42 (4.29) |
| PNI | 49 (5.01) |
| Neutrophil count | 24 (2.45) |
| Lymphocyte count | 24 (2.45) |
| Monocyte count | 24 (2.45) |
| Platelet count | 22 (2.25) |
| Albumin | 45 (4.60) |
| HDL cholesterol | 40 (4.09) |

Missing values are presented as n (%) among the 978 participants in the prediction sample. Clinical predictors not listed had no missing values.

**Supplementary Table S3.** Subgroup analyses of all-cause mortality comparing DM + P with P.

| Subgroup | Category | Deaths/participants | HR (95% CI) | P value | P for interaction |
|---|---|---|---|---|---|
| Sex | Female | 72/398 vs 138/1,438 | 1.73 (1.31–2.29) | <0.001 | 0.030 |
| | Male | 130/580 vs 238/2,037 | 1.10 (0.85–1.42) | 0.479 | |
| Age | <65 years | 71/583 vs 131/2,533 | 2.03 (1.34–3.09) | <0.001 | 0.037 |
| | ≥65 years | 131/395 vs 245/942 | 1.14 (0.89–1.45) | 0.302 | |
| Race/ethnicity | Mexican American | 35/208 vs 25/542 | 3.23 (1.77–5.90) | <0.001 | 0.056 |
| | Non-Hispanic Black | 58/284 vs 79/838 | 1.44 (0.93–2.22) | 0.103 | |
| | Non-Hispanic White | 86/284 vs 240/1,376 | 1.22 (0.95–1.58) | 0.123 | |
| | Other Hispanic | 15/95 vs 17/317 | 1.45 (0.46–4.52) | 0.525 | |
| | Other race | 8/107 vs 15/402 | 0.54 (0.14–2.04) | 0.368 | |
| Smoking status | Ever smoker | 121/512 vs 227/1,912 | 1.45 (1.15–1.84) | 0.002 | 0.210 |
| | Never smoker | 81/466 vs 149/1,563 | 1.11 (0.80–1.53) | 0.536 | |
| BMI | <25 kg/m² | 34/130 vs 161/1,035 | 1.15 (0.80–1.67) | 0.452 | 0.251 |
| | 25–<30 kg/m² | 71/293 vs 109/1,249 | 1.79 (1.23–2.59) | 0.002 | |
| | ≥30 kg/m² | 97/555 vs 106/1,191 | 1.11 (0.72–1.71) | 0.648 | |

Deaths/participants are presented as DM + P vs P. HRs and 95% CIs were estimated using fully adjusted survey-weighted Cox proportional hazards models accounting for the 6-year NHANES examination weights, sampling strata, and primary sampling units. P for interaction was obtained from the interaction term between DM–periodontitis status and each subgroup variable. HR, hazard ratio; CI, confidence interval; BMI, body mass index. Group abbreviations are defined in Table 1 of the main manuscript.

**Supplementary Table S4.** Subgroup analyses of all-cause mortality comparing DM + P with DM.

| Subgroup | Category | Deaths/participants | HR (95% CI) | P value | P for interaction |
|---|---|---|---|---|---|
| Sex | Female | 72/398 vs 84/504 | 1.19 (0.79–1.81) | 0.403 | 0.298 |
| | Male | 130/580 vs 96/393 | 0.90 (0.62–1.32) | 0.587 | |
| Age | <65 years | 71/583 vs 46/515 | 1.05 (0.67–1.66) | 0.826 | 0.629 |
| | ≥65 years | 131/395 vs 134/382 | 0.91 (0.62–1.34) | 0.638 | |
| Race/ethnicity | Mexican American | 35/208 vs 7/87 | 2.48 (1.20–5.15) | 0.014 | 0.069 |
| | Non-Hispanic Black | 58/284 vs 43/222 | 1.23 (0.81–1.87) | 0.327 | |
| | Non-Hispanic White | 86/284 vs 107/397 | 0.95 (0.65–1.40) | 0.809 | |
| | Other Hispanic | 15/95 vs 10/97 | 1.18 (0.42–3.32) | 0.755 | |
| | Other race | 8/107 vs 13/94 | 0.22 (0.07–0.76) | 0.016 | |
| Smoking status | Ever smoker | 121/512 vs 114/435 | 1.08 (0.78–1.48) | 0.649 | 0.466 |
| | Never smoker | 81/466 vs 66/462 | 0.87 (0.51–1.49) | 0.607 | |
| BMI | <25 kg/m² | 34/130 vs 38/117 | 0.70 (0.40–1.23) | 0.217 | 0.250 |
| | 25–<30 kg/m² | 71/293 vs 51/234 | 1.34 (0.81–2.24) | 0.256 | |
| | ≥30 kg/m² | 97/555 vs 91/546 | 0.92 (0.61–1.39) | 0.704 | |

Deaths/participants are presented as DM + P vs DM. HRs and 95% CIs were estimated using fully adjusted survey-weighted Cox proportional hazards models accounting for the 6-year NHANES examination weights, sampling strata, and primary sampling units. P for interaction was obtained from the interaction term between DM–periodontitis status and each subgroup variable. HR, hazard ratio; CI, confidence interval; BMI, body mass index. Group abbreviations are defined in Table 1 of the main manuscript.

**Supplementary Table S5.** Subgroup analyses of cardiovascular mortality comparing DM + P with P.

| Subgroup | Category | Deaths/participants | HR (95% CI) | P value | P for interaction |
|---|---|---|---|---|---|
| Sex | Female | 21/398 vs 36/1,438 | 2.05 (1.17–3.58) | 0.012 | 0.333 |
| | Male | 47/580 vs 69/2,037 | 1.36 (0.85–2.19) | 0.199 | |
| Age | <65 years | 21/583 vs 23/2,533 | 4.15 (1.76–9.81) | 0.001 | 0.013 |
| | ≥65 years | 47/395 vs 82/942 | 1.16 (0.81–1.67) | 0.414 | |
| Race/ethnicity | Mexican American | 9/208 vs 6/542 | 3.76 (1.09–12.97) | 0.036 | 0.473 |
| | Non-Hispanic Black | 18/284 vs 26/838 | 1.04 (0.45–2.39) | 0.920 | |
| | Non-Hispanic White | 33/284 vs 65/1,376 | 1.66 (1.08–2.55) | 0.021 | |
| | Other Hispanic | 5/95 vs 3/317 | 1.86 (0.31–11.08) | 0.497 | |
| | Other race | 3/107 vs 5/402 | 0.73 (0.12–4.59) | 0.738 | |
| Smoking status | Ever smoker | 32/512 vs 56/1,912 | 1.74 (1.21–2.50) | 0.003 | 0.511 |
| | Never smoker | 36/466 vs 49/1,563 | 1.44 (0.89–2.33) | 0.141 | |
| BMI | <25 kg/m² | 10/130 vs 41/1,035 | 1.14 (0.51–2.59) | 0.747 | 0.614 |
| | 25–<30 kg/m² | 23/293 vs 39/1,249 | 1.77 (0.90–3.52) | 0.100 | |
| | ≥30 kg/m² | 35/555 vs 25/1,191 | 1.98 (1.02–3.84) | 0.045 | |

Deaths/participants are presented as DM + P vs P. Cause-specific HRs and 95% CIs were estimated using fully adjusted survey-weighted Cox proportional hazards models, with noncardiovascular deaths censored. Models accounted for the 6-year NHANES examination weights, sampling strata, and primary sampling units. P for interaction was obtained from the interaction term between DM–periodontitis status and each subgroup variable. HR, hazard ratio; CI, confidence interval; BMI, body mass index. Group abbreviations are defined in Table 1 of the main manuscript.

**Supplementary Table S6.** Subgroup analyses of cardiovascular mortality comparing DM + P with DM.

| Subgroup | Category | Deaths/participants | HR (95% CI) | P value | P for interaction |
|---|---|---|---|---|---|
| Sex | Female | 21/398 vs 29/504 | 1.22 (0.60–2.44) | 0.584 | 0.655 |
| | Male | 47/580 vs 32/393 | 1.04 (0.64–1.68) | 0.878 | |
| Age | <65 years | 21/583 vs 14/515 | 1.07 (0.32–3.59) | 0.911 | 0.911 |
| | ≥65 years | 47/395 vs 47/382 | 0.99 (0.60–1.65) | 0.970 | |
| Race/ethnicity | Mexican American | 9/208 vs 2/87 | 3.08 (0.70–13.64) | 0.138 | 0.223 |
| | Non-Hispanic Black | 18/284 vs 13/222 | 1.57 (0.67–3.70) | 0.302 | |
| | Non-Hispanic White | 33/284 vs 39/397 | 1.08 (0.58–2.04) | 0.802 | |
| | Other Hispanic | 5/95 vs 1/97 | 3.20 (0.35–29.50) | 0.304 | |
| | Other race | 3/107 vs 6/94 | 0.20 (0.04–1.06) | 0.059 | |
| Smoking status | Ever smoker | 32/512 vs 39/435 | 0.99 (0.59–1.64) | 0.954 | 0.512 |
| | Never smoker | 36/466 vs 22/462 | 1.29 (0.60–2.78) | 0.514 | |
| BMI | <25 kg/m² | 10/130 vs 13/117 | 0.68 (0.26–1.80) | 0.443 | 0.587 |
| | 25–<30 kg/m² | 23/293 vs 16/234 | 1.32 (0.58–2.99) | 0.508 | |
| | ≥30 kg/m² | 35/555 vs 32/546 | 1.10 (0.56–2.15) | 0.774 | |

Deaths/participants are presented as DM + P vs DM. Cause-specific HRs and 95% CIs were estimated using fully adjusted survey-weighted Cox proportional hazards models, with noncardiovascular deaths censored. Models accounted for the 6-year NHANES examination weights, sampling strata, and primary sampling units. P for interaction was obtained from the interaction term between DM–periodontitis status and each subgroup variable. HR, hazard ratio; CI, confidence interval; BMI, body mass index. Group abbreviations are defined in Table 1 of the main manuscript.

**Supplementary Table S7.** Multiplicative interaction between DM and periodontitis in relation to all-cause and cardiovascular mortality.

| Outcome | Model | P HR (95% CI) | DM HR (95% CI) | DM + P HR (95% CI) | Interaction ratio (95% CI) | P for interaction |
|---|---|---|---|---|---|---|
| All-cause mortality | Model 1 | 1.98 (1.62–2.42) | 3.66 (2.87–4.66) | 3.71 (2.91–4.72) | 0.51 (0.38–0.70) | <0.001 |
| All-cause mortality | Model 2 | 1.21 (0.99–1.47) | 1.67 (1.29–2.15) | 1.65 (1.28–2.13) | 0.82 (0.60–1.13) | 0.230 |
| All-cause mortality | Model 3 | 1.15 (0.95–1.40) | 1.51 (1.17–1.95) | 1.51 (1.17–1.94) | 0.87 (0.63–1.19) | 0.378 |
| Cardiovascular mortality | Model 1 | 1.70 (1.25–2.32) | 4.11 (2.88–5.86) | 4.56 (3.14–6.61) | 0.65 (0.43–0.98) | 0.047 |
| Cardiovascular mortality | Model 2 | 0.95 (0.70–1.30) | 1.60 (1.11–2.31) | 1.77 (1.22–2.58) | 1.16 (0.77–1.75) | 0.473 |
| Cardiovascular mortality | Model 3 | 0.96 (0.71–1.30) | 1.40 (0.98–2.00) | 1.52 (1.00–2.32) | 1.13 (0.74–1.74) | 0.575 |

HRs and 95% CIs were estimated using survey-weighted Cox proportional hazards models including binary indicators for DM and periodontitis and their product term. The interaction ratio represents the HR for DM + P divided by the product of the individual HRs for DM and P. P for interaction was obtained from a design-based Wald test of the product term for DM and periodontitis. For cardiovascular mortality, cause-specific HRs were estimated with noncardiovascular deaths censored. Model 1 was unadjusted. Model 2 adjusted for age, sex, race/ethnicity, and educational attainment. Model 3 additionally adjusted for poverty-to-income ratio, body mass index, smoking status, and hypertension. HR, hazard ratio; CI, confidence interval; DM, diabetes mellitus; P, periodontitis.

**Supplementary Table S8.** Landmark analyses of mortality by DM–periodontitis status.

| Analysis | Outcome | Group | Deaths/participants | HR (95% CI) | P value |
|---|---|---|---|---|---|
| Original Cox analysis | All-cause mortality | NDM + NP | 400/5,282 | 1.00 (Reference) | — |
| Original Cox analysis | All-cause mortality | P | 376/3,475 | 1.15 (0.95–1.40) | 0.149 |
| Original Cox analysis | All-cause mortality | DM | 180/897 | 1.51 (1.17–1.95) | 0.001 |
| Original Cox analysis | All-cause mortality | DM + P | 202/978 | 1.51 (1.17–1.94) | 0.001 |
| Original Cox analysis | Cardiovascular mortality | NDM + NP | 117/5,282 | 1.00 (Reference) | — |
| Original Cox analysis | Cardiovascular mortality | P | 105/3,475 | 0.96 (0.71–1.30) | 0.782 |
| Original Cox analysis | Cardiovascular mortality | DM | 61/897 | 1.40 (0.98–2.00) | 0.061 |
| Original Cox analysis | Cardiovascular mortality | DM + P | 68/978 | 1.52 (1.00–2.32) | 0.051 |
| 1-year landmark analysis | All-cause mortality | NDM + NP | 372/5,254 | 1.00 (Reference) | — |
| 1-year landmark analysis | All-cause mortality | P | 344/3,443 | 1.13 (0.93–1.38) | 0.226 |
| 1-year landmark analysis | All-cause mortality | DM | 174/891 | 1.57 (1.20–2.06) | 0.001 |
| 1-year landmark analysis | All-cause mortality | DM + P | 185/961 | 1.51 (1.15–1.99) | 0.003 |
| 1-year landmark analysis | Cardiovascular mortality | NDM + NP | 111/5,254 | 1.00 (Reference) | — |
| 1-year landmark analysis | Cardiovascular mortality | P | 97/3,443 | 0.96 (0.70–1.31) | 0.793 |
| 1-year landmark analysis | Cardiovascular mortality | DM | 57/891 | 1.40 (0.97–2.04) | 0.076 |
| 1-year landmark analysis | Cardiovascular mortality | DM + P | 63/961 | 1.51 (0.98–2.31) | 0.060 |
| 2-year landmark analysis | All-cause mortality | NDM + NP | 325/5,207 | 1.00 (Reference) | — |
| 2-year landmark analysis | All-cause mortality | P | 310/3,409 | 1.16 (0.95–1.41) | 0.136 |
| 2-year landmark analysis | All-cause mortality | DM | 158/875 | 1.64 (1.24–2.15) | <0.001 |
| 2-year landmark analysis | All-cause mortality | DM + P | 162/938 | 1.53 (1.14–2.05) | 0.004 |
| 2-year landmark analysis | Cardiovascular mortality | NDM + NP | 96/5,207 | 1.00 (Reference) | — |
| 2-year landmark analysis | Cardiovascular mortality | P | 88/3,409 | 1.03 (0.74–1.43) | 0.854 |
| 2-year landmark analysis | Cardiovascular mortality | DM | 56/875 | 1.66 (1.13–2.44) | 0.010 |
| 2-year landmark analysis | Cardiovascular mortality | DM + P | 57/938 | 1.62 (1.03–2.55) | 0.037 |

Deaths/participants indicate the number of outcome-specific deaths and participants included in each analysis. Landmark analyses included participants who remained alive at the corresponding 1- or 2-year landmark, with follow-up beginning from the landmark time. HRs and 95% CIs were estimated using fully adjusted survey-weighted Cox proportional hazards models accounting for the 6-year NHANES examination weights, sampling strata, and primary sampling units. For cardiovascular mortality, cause-specific HRs were estimated with noncardiovascular deaths censored. HR, hazard ratio; CI, confidence interval. Group abbreviations are defined in Table 1 of the main manuscript.

**Supplementary Table S9.** Landmark analyses of mortality comparing DM + P with either condition alone.

| Analysis | Outcome | Comparison | Deaths/participants (DM + P/reference) | HR (95% CI) | P value |
|---|---|---|---|---|---|
| Original Cox analysis | All-cause mortality | DM + P vs P | 202/978 vs 376/3,475 | 1.31 (1.09–1.57) | 0.003 |
| Original Cox analysis | All-cause mortality | DM + P vs DM | 202/978 vs 180/897 | 1.00 (0.73–1.35) | 0.984 |
| Original Cox analysis | Cardiovascular mortality | DM + P vs P | 68/978 vs 105/3,475 | 1.59 (1.16–2.18) | 0.004 |
| Original Cox analysis | Cardiovascular mortality | DM + P vs DM | 68/978 vs 61/897 | 1.08 (0.67–1.76) | 0.741 |
| 1-year landmark analysis | All-cause mortality | DM + P vs P | 185/961 vs 344/3,443 | 1.33 (1.09–1.63) | 0.005 |
| 1-year landmark analysis | All-cause mortality | DM + P vs DM | 185/961 vs 174/891 | 0.96 (0.70–1.32) | 0.809 |
| 1-year landmark analysis | Cardiovascular mortality | DM + P vs P | 63/961 vs 97/3,443 | 1.57 (1.11–2.23) | 0.012 |
| 1-year landmark analysis | Cardiovascular mortality | DM + P vs DM | 63/961 vs 57/891 | 1.07 (0.66–1.74) | 0.768 |
| 2-year landmark analysis | All-cause mortality | DM + P vs P | 162/938 vs 310/3,409 | 1.32 (1.07–1.62) | 0.009 |
| 2-year landmark analysis | All-cause mortality | DM + P vs DM | 162/938 vs 158/875 | 0.94 (0.66–1.32) | 0.703 |
| 2-year landmark analysis | Cardiovascular mortality | DM + P vs P | 57/938 vs 88/3,409 | 1.57 (1.10–2.25) | 0.014 |
| 2-year landmark analysis | Cardiovascular mortality | DM + P vs DM | 57/938 vs 56/875 | 0.98 (0.60–1.58) | 0.920 |

Deaths/participants are presented as DM + P vs the corresponding reference group. Landmark analyses included participants who remained alive at the corresponding 1- or 2-year landmark, with follow-up beginning from the landmark time. HRs and 95% CIs were estimated using fully adjusted survey-weighted Cox proportional hazards models accounting for the 6-year NHANES examination weights, sampling strata, and primary sampling units. For cardiovascular mortality, cause-specific HRs were estimated with noncardiovascular deaths censored. HR, hazard ratio; CI, confidence interval. Group abbreviations are defined in Table 1 of the main manuscript.

**Supplementary Table S10.** Complete-case and multiple-imputation estimates for mortality associations according to DM–periodontitis status.

| Outcome | Comparison | Complete-case estimate (95% CI) | Multiple-imputation estimate (95% CI) |
|---|---|---|---|
| All-cause mortality | P vs NDM + NP | 1.15 (0.95–1.40) | 1.14 (0.94–1.39) |
| | DM vs NDM + NP | 1.51 (1.17–1.95) | 1.55 (1.19–2.02) |
| | DM + P vs NDM + NP | 1.51 (1.17–1.94) | 1.45 (1.13–1.85) |
| | DM + P vs P | 1.31 (1.09–1.57) | 1.27 (1.06–1.51) |
| | DM + P vs DM | 1.00 (0.73–1.35) | 0.93 (0.69–1.26) |
| Cardiovascular mortality | P vs NDM + NP | 0.98 (0.72–1.33) | 0.96 (0.72–1.27) |
| | DM vs NDM + NP | 1.34 (0.95–1.90) | 1.45 (1.04–2.04) |
| | DM + P vs NDM + NP | 1.51 (1.003–2.276) | 1.43 (0.97–2.10) |
| | DM + P vs P | 1.55 (1.14–2.10) | 1.49 (1.13–1.95) |
| | DM + P vs DM | 1.13 (0.71–1.79) | 0.98 (0.66–1.45) |

Complete-case estimates were obtained from the primary Model 3 analyses among 10,632 participants. Multiple-imputation estimates were obtained from 20 imputed datasets including all 11,734 eligible participants and were pooled using Rubin's rules. All-cause mortality estimates are reported as HRs and cardiovascular mortality estimates as sHRs. Model 3 adjusted for age, sex, race/ethnicity, educational attainment, poverty-to-income ratio, body mass index, smoking status, and hypertension. HR, hazard ratio; sHR, subdistribution hazard ratio; CI, confidence interval; NDM + NP, neither diabetes mellitus nor moderate-to-severe periodontitis; P, moderate-to-severe periodontitis alone; DM, diabetes mellitus without moderate-to-severe periodontitis; DM + P, concurrent diabetes mellitus and moderate-to-severe periodontitis.

**Supplementary Table S11.** Diabetes-specific sensitivity analyses for mortality comparing DM + P with DM.

| Analysis | Outcome | N (events) | Model 3 estimate (95% CI) | Additionally adjusted estimate (95% CI) |
|---|---|---|---|---|
| HbA1c-complete subset | All-cause mortality | 1,830 (363) | 1.05 (0.76–1.45) | 1.04 (0.75–1.44) |
| | Cardiovascular mortality | 1,830 (125) | 1.20 (0.74–1.95) | 1.19 (0.74–1.93) |
| Physician-diagnosed diabetes complete subset | All-cause mortality | 1,396 (295) | 1.08 (0.78–1.50) | 1.07 (0.76–1.49) |
| | Cardiovascular mortality | 1,396 (108) | 1.17 (0.70–1.94) | 1.18 (0.67–2.06) |

N and events are unweighted counts. All estimates compare DM + P with DM and are reported as HRs for all-cause mortality and sHRs for cardiovascular mortality. Model 3 adjusted for age, sex, race/ethnicity, educational attainment, poverty-to-income ratio, body mass index, smoking status, and hypertension. The additionally adjusted model included HbA1c in the HbA1c-complete subset and HbA1c, diabetes duration, current insulin use, and current use of diabetes pills in the physician-diagnosed diabetes complete subset. HR, hazard ratio; sHR, subdistribution hazard ratio; CI, confidence interval; DM, diabetes mellitus without moderate-to-severe periodontitis; DM + P, concurrent diabetes mellitus and moderate-to-severe periodontitis.

**Supplementary Table S12.** Periodontitis-severity sensitivity analysis for all-cause and cardiovascular mortality.

| Group or comparison | Participants (all-cause/CVD deaths) | All-cause HR (95% CI) | Cardiovascular sHR (95% CI) |
|---|---|---|---|
| **Six-group estimates versus NDM + NP** | | | |
| NDM + NP | 5,282 (400/117) | 1.00 (Reference) | 1.00 (Reference) |
| Moderate P | 2,668 (279/80) | 1.10 (0.88–1.38); P = 0.388 | 1.00 (0.72–1.40); P = 0.986 |
| Severe P | 807 (97/25) | 1.37 (0.96–1.94); P = 0.081 | 0.87 (0.53–1.42); P = 0.568 |
| DM | 897 (180/61) | 1.51 (1.17–1.95); P = 0.001 | 1.34 (0.95–1.90); P = 0.097 |
| DM + moderate P | 729 (156/52) | 1.52 (1.17–1.97); P = 0.002 | 1.43 (0.88–2.34); P = 0.149 |
| DM + severe P | 249 (46/16) | 1.49 (1.01–2.22); P = 0.046 | 1.84 (0.90–3.75); P = 0.094 |
| **Key pairwise comparisons** | | | |
| severe P vs moderate P | — | 1.24 (0.82–1.86); P = 0.302 | 0.86 (0.50–1.50); P = 0.604 |
| DM + severe P vs DM + moderate P | — | 0.99 (0.68–1.43); P = 0.937 | 1.28 (0.54–3.06); P = 0.576 |
| DM + moderate P vs moderate P | — | 1.38 (1.14–1.66); P < 0.001 | 1.43 (1.02–2.01); P = 0.040 |
| DM + severe P vs severe P | — | 1.09 (0.71–1.68); P = 0.684 | 2.12 (0.98–4.61); P = 0.057 |
| DM + moderate P vs DM | — | 1.00 (0.74–1.37); P = 0.986 | 1.07 (0.63–1.82); P = 0.805 |
| DM + severe P vs DM | — | 0.99 (0.64–1.53); P = 0.956 | 1.37 (0.65–2.89); P = 0.408 |

Participants and deaths are unweighted counts; values in parentheses indicate all-cause and cardiovascular deaths, respectively. Model 3 adjusted for age, sex, race/ethnicity, educational attainment, poverty-to-income ratio, body mass index, smoking status, and hypertension. HR, hazard ratio; sHR, subdistribution hazard ratio; CI, confidence interval; CVD, cardiovascular; NDM + NP, neither diabetes mellitus nor moderate-to-severe periodontitis; P, periodontitis; DM, diabetes mellitus without moderate-to-severe periodontitis.

**Supplementary Table S13.** Extended-covariate sensitivity analyses for all-cause and cardiovascular mortality.

| Outcome | Comparison | Restricted-sample Model 3 | Extended-1 | Extended-2 |
|---|---|---|---|---|
| All-cause mortality | DM + P vs NDM + NP | 1.53 (1.18–1.99); P = 0.001 | 1.45 (1.11–1.89); P = 0.007 | 1.26 (0.97–1.63); P = 0.085 |
| | DM + P vs P | 1.28 (1.06–1.55); P = 0.009 | 1.19 (0.97–1.46); P = 0.104 | 1.03 (0.82–1.28); P = 0.820 |
| | DM + P vs DM | 1.03 (0.73–1.46); P = 0.868 | 1.04 (0.73–1.47); P = 0.822 | 1.14 (0.81–1.61); P = 0.457 |
| Cardiovascular mortality | DM + P vs NDM + NP | 1.61 (1.05–2.47); P = 0.028 | 1.53 (0.99–2.37); P = 0.055 | 1.29 (0.82–2.02); P = 0.264 |
| | DM + P vs P | 1.64 (1.15–2.35); P = 0.007 | 1.57 (1.06–2.32); P = 0.024 | 1.35 (0.92–1.98); P = 0.120 |
| | DM + P vs DM | 1.23 (0.72–2.12); P = 0.454 | 1.23 (0.72–2.09); P = 0.450 | 1.48 (0.85–2.56); P = 0.164 |

All analyses used the same common complete-case subset of 9,358 participants. All-cause mortality estimates are reported as HRs and cardiovascular mortality estimates as sHRs. Restricted-sample Model 3 adjusted for age, sex, race/ethnicity, educational attainment, poverty-to-income ratio, body mass index, smoking status, and hypertension. Extended-1 additionally adjusted for total cholesterol, alcohol exposure, physical activity, usual source of care, and health insurance status. Extended-2 further adjusted for established cardiovascular disease, reduced estimated glomerular filtration rate, and albuminuria. HR, hazard ratio; sHR, subdistribution hazard ratio; CI, confidence interval; NDM + NP, neither diabetes mellitus nor moderate-to-severe periodontitis; P, moderate-to-severe periodontitis alone; DM, diabetes mellitus without moderate-to-severe periodontitis; DM + P, concurrent diabetes mellitus and moderate-to-severe periodontitis.

**Supplementary Table S14.** Internally validated performance of the nine prespecified candidate models.

| Model | Harrell C-index (95% CI) | 3-year AUC (95% CI) | 3-year Brier score (95% CI) | 5-year AUC (95% CI) | 5-year Brier score (95% CI) | 5-year calibration intercept (95% CI) | 5-year calibration slope (95% CI) |
|---|---|---|---|---|---|---|---|
| Clinical elastic-net Cox | 0.719 (0.680–0.755) | 0.657 (0.582–0.731) | 0.055 (0.043–0.069) | 0.725 (0.669–0.779) | 0.094 (0.080–0.108) | 0.004 (−0.194–0.170) | 6.340 (4.564–8.366) |
| Inflammation elastic-net Cox | 0.628 (0.587–0.670) | 0.606 (0.522–0.678) | 0.055 (0.043–0.069) | 0.602 (0.542–0.665) | 0.095 (0.081–0.110) | 0.002 (−0.193–0.171) | 2.513 (0.953–4.105) |
| Combined elastic-net Cox | 0.723 (0.685–0.760) | 0.669 (0.593–0.740) | 0.055 (0.042–0.068) | 0.734 (0.680–0.784) | 0.091 (0.078–0.106) | 0.000 (−0.193–0.169) | 2.566 (1.927–3.251) |
| Clinical random survival forest | 0.728 (0.692–0.766) | 0.621 (0.546–0.695) | 0.055 (0.043–0.069) | 0.724 (0.668–0.778) | 0.087 (0.074–0.100) | −0.021 (−0.215–0.158) | 1.133 (0.855–1.409) |
| Inflammation random survival forest | 0.669 (0.632–0.708) | 0.652 (0.570–0.724) | 0.054 (0.043–0.066) | 0.660 (0.606–0.712) | 0.093 (0.080–0.107) | −0.046 (−0.245–0.129) | 0.715 (0.438–0.990) |
| Combined random survival forest | 0.742 (0.707–0.779) | 0.669 (0.596–0.739) | 0.055 (0.043–0.067) | 0.734 (0.683–0.788) | 0.087 (0.074–0.101) | −0.049 (−0.247–0.126) | 0.920 (0.687–1.181) |
| Clinical XGBoost Cox | 0.739 (0.704–0.772) | 0.659 (0.589–0.727) | 0.056 (0.044–0.069) | 0.740 (0.687–0.792) | 0.085 (0.071–0.098) | 0.014 (−0.182–0.197) | 1.008 (0.802–1.230) |
| Inflammation XGBoost Cox | 0.660 (0.621–0.700) | 0.648 (0.575–0.717) | 0.055 (0.043–0.068) | 0.645 (0.590–0.699) | 0.094 (0.080–0.108) | −0.012 (−0.210–0.159) | 0.827 (0.503–1.166) |
| Combined XGBoost Cox | 0.754 (0.721–0.788) | 0.690 (0.618–0.757) | 0.055 (0.043–0.068) | 0.754 (0.702–0.804) | 0.085 (0.072–0.098) | 0.025 (−0.171–0.199) | 0.968 (0.775–1.171) |

Performance was evaluated using participant-averaged out-of-fold predictions from five repeats of fivefold outer cross-validation. Harrell's C-index summarizes discrimination over the available follow-up. Time-dependent AUC and Brier scores were estimated at 3 and 5 years using inverse-probability-of-censoring weighting. Calibration intercepts and slopes were estimated at 5 years using inverse-probability-of-censoring-weighted complementary log–log regression. Values are presented with 95% confidence intervals obtained from 1,000 participant-level bootstrap resamples of the final out-of-fold prediction dataset. AUC, area under the time-dependent receiver operating characteristic curve; CI, confidence interval.

**Supplementary Table S15.** Paired comparison of the Clinical and Combined XGBoost Cox models.

| Metric | Clinical model | Combined model (Clinical + composite indices) | Paired difference* | 95% CI | Bootstrap P value |
|---|---|---|---|---|---|
| Harrell C-index | 0.739 (0.704–0.772) | 0.754 (0.721–0.788) | 0.015 | −0.006 to 0.036 | 0.164 |
| 5-year AUC | 0.740 (0.687–0.792) | 0.754 (0.702–0.804) | 0.014 | −0.017 to 0.043 | 0.370 |
| 5-year Brier score | 0.0848 (0.0714–0.0983) | 0.0851 (0.0720–0.0985) | −0.0003 | −0.0028 to 0.0022 | 0.813 |
| 5-year calibration intercept | 0.014 (−0.182–0.197) | 0.025 (−0.171–0.199) | −0.011 | −0.044 to 0.041 | 0.947 |
| 5-year calibration slope | 1.008 (0.802–1.230) | 0.968 (0.775–1.171) | −0.024 | −0.110 to 0.122 | 0.997 |

The Combined model included the Clinical predictors and eight composite inflammatory indices. Paired differences were calculated from participant-averaged out-of-fold predictions. For the C-index and AUC, differences are Combined minus Clinical; for the Brier score, the difference is Clinical minus Combined. For calibration, differences compare the absolute distance from the ideal value (0 for the intercept and 1 for the slope), with positive values favoring the Combined model. Confidence intervals and two-sided P values were obtained from 1,000 paired participant-level bootstrap resamples of the final out-of-fold prediction dataset; models were not refitted during bootstrap resampling. AUC, area under the time-dependent receiver operating characteristic curve; CI, confidence interval.

**Supplementary Table S16.** Verification of 5-year calibration slopes and prediction dispersion.

| Model | Reported / recalculated slope | Repeat-specific slopes (1–5) | Alternative aggregation slopes* | Predicted-risk dispersion† |
|---|---|---|---|---|
| Clinical elastic-net Cox | 6.34 / 6.34 | 8.28, 4.88, 2.64, 8.19, 8.33 | 6.45 / 6.31 | 0.013 / 0.017 |
| Inflammation elastic-net Cox | 2.51 / 2.51 | 1.92, 2.46, 0.70, 1.53, 3.50 | 2.67 / 2.45 | 0.012 / 0.010 |
| Combined elastic-net Cox | 2.57 / 2.57 | 1.24, 1.93, 2.16, 1.63, 3.35 | 2.62 / 2.51 | 0.031 / 0.035 |
| Clinical Cox benchmark | 0.89 / 0.89 | 0.90, 0.86, 0.84, 0.87, 0.91 | 0.89 / 0.89 | 0.096 / 0.102 |

*Values are complementary log–log-scale and cumulative-hazard-scale aggregation slopes, respectively. †Values are the SD and IQR, respectively, of participant-level predicted risks obtained using the prespecified arithmetic mean of absolute risks across the five repeats.

**Supplementary Table S17.** Internally validated performance of the conventional Clinical Cox benchmark and the partially penalized Combined Cox sensitivity model.

| Model | Metric | Estimate (95% CI) |
|---|---|---|
| Clinical Cox | Harrell C-index | 0.729 (0.692–0.764) |
| Clinical Cox | 3-year AUC | 0.647 (0.577–0.718) |
| Clinical Cox | 3-year Brier score | 0.056 (0.044–0.069) |
| Clinical Cox | 5-year AUC | 0.734 (0.679–0.785) |
| Clinical Cox | 5-year Brier score | 0.087 (0.074–0.100) |
| Clinical Cox | 5-year calibration intercept | 0.013 (−0.178–0.191) |
| Clinical Cox | 5-year calibration slope | 0.892 (0.643–1.153) |
| Combined partially penalized Cox | Harrell C-index | 0.727 (0.691–0.763) |
| Combined partially penalized Cox | 3-year AUC | 0.646 (0.576–0.717) |
| Combined partially penalized Cox | 3-year Brier score | 0.056 (0.044–0.069) |
| Combined partially penalized Cox | 5-year AUC | 0.734 (0.680–0.784) |
| Combined partially penalized Cox | 5-year Brier score | 0.087 (0.074–0.100) |
| Combined partially penalized Cox | 5-year calibration intercept | 0.012 (−0.181–0.190) |
| Combined partially penalized Cox | 5-year calibration slope | 0.909 (0.662–1.174) |

Performance was evaluated using participant-averaged out-of-fold predictions from five repeats of fivefold outer cross-validation. Harrell's C-index summarizes discrimination over the available follow-up. Time-dependent AUC and Brier scores were estimated at 3 and 5 years using inverse-probability-of-censoring weighting. Calibration intercepts and slopes were estimated at 5 years using inverse-probability-of-censoring-weighted complementary log–log regression. The partially penalized Combined Cox model included the Clinical predictors without penalization and the eight composite inflammatory indices with penalization. Values are presented with 95% confidence intervals obtained from 1,000 participant-level bootstrap resamples of the final out-of-fold prediction dataset; models were not refitted during bootstrap resampling. AUC, area under the time-dependent receiver operating characteristic curve; CI, confidence interval.

**Supplementary Table S18.** Unweighted and examination-weighted performance of the Clinical and Combined XGBoost Cox models.

| Specification | Metric | Clinical | Combined | Paired difference* | 95% CI | Bootstrap P value |
|---|---|---|---|---|---|---|
| Unweighted | Harrell C-index | 0.7386 | 0.7538 | 0.0152 | −0.0058 to 0.0358 | 0.164 |
| Unweighted | 3-year AUC | 0.6594 | 0.6898 | 0.0304 | −0.0098 to 0.0732 | 0.140 |
| Unweighted | 3-year Brier score | 0.0557 | 0.0554 | 0.0003 | −0.0011 to 0.0017 | 0.641 |
| Unweighted | 5-year AUC | 0.7401 | 0.7536 | 0.0135 | −0.0170 to 0.0428 | 0.370 |
| Unweighted | 5-year Brier score | 0.0848 | 0.0851 | −0.0003 | −0.0028 to 0.0022 | 0.813 |
| MEC examination-weighted | Harrell C-index | 0.7340 | 0.7540 | 0.0200 | −0.0049 to 0.0466 | 0.134 |
| MEC examination-weighted | 3-year AUC | 0.6657 | 0.7080 | 0.0424 | −0.0055 to 0.0941 | 0.090 |
| MEC examination-weighted | 3-year Brier score | 0.0476 | 0.0478 | −0.0001 | −0.0014 to 0.0011 | 0.835 |
| MEC examination-weighted | 5-year AUC | 0.7308 | 0.7444 | 0.0135 | −0.0237 to 0.0546 | 0.503 |
| MEC examination-weighted | 5-year Brier score | 0.0757 | 0.0760 | −0.0002 | −0.0025 to 0.0020 | 0.803 |

Unweighted estimates correspond to the primary prediction analysis. Examination-weighted estimates used normalized 6-year NHANES examination weights with a mean of 1. *For the C-index and AUC, paired differences were calculated as Combined minus Clinical; for the Brier score, paired differences were calculated as Clinical minus Combined. Positive differences therefore favor the Combined model. Confidence intervals and two-sided P values were obtained from 1,000 paired participant-level bootstrap resamples of the final out-of-fold prediction dataset; models were not refitted during bootstrap resampling. The examination-weighted analysis used weighted censoring estimation and weighted aggregation of performance measures but did not incorporate sampling strata or primary sampling units. AUC, area under the time-dependent receiver operating characteristic curve; CI, confidence interval; MEC, Mobile Examination Center.

**Supplementary Table S19.** Descriptive risk stratification based on out-of-fold 5-year mortality risk.

| Predicted-risk tertile | Participants (deaths) | Observed 3-year risk (95% CI) | Observed 5-year risk (95% CI) | Median follow-up, y | Median predicted 5-year risk | HR vs lowest tertile (95% CI) |
|---|---|---|---|---|---|---|
| Lowest | 326 (20) | 2.5% (1.2%–4.8%) | 3.7% (2.1%–6.4%) | 8.08 | 0.035 | 1.00 (Reference); — |
| Middle | 326 (44) | 4.9% (3.0%–7.9%) | 7.4% (5.0%–10.8%) | 7.67 | 0.060 | 2.26 (1.33–3.83); P = 0.003 |
| Highest | 326 (138) | 10.4% (7.6%–14.3%) | 21.2% (17.1%–26.0%) | 6.46 | 0.177 | 8.59 (5.37–13.73); P < 0.001 |

Predicted-risk tertiles were defined as equal-frequency tertiles of participant-averaged out-of-fold 5-year mortality risk from the Combined XGBoost Cox model. Observed 3- and 5-year mortality risks were estimated using Kaplan–Meier methods. HRs and 95% CIs were estimated using unadjusted, unweighted Cox proportional hazards models with the lowest tertile as the reference. The overall survival difference was assessed using an unweighted log-rank test ($P < 0.001$). Tertile cut points were derived from the present study sample and were not selected to optimize outcome separation. These tertiles were descriptive and were not intended as clinical treatment thresholds. HR, hazard ratio; CI, confidence interval.

## 3. Supplementary Figures

**DM + P vs P**

| Subgroup | Category | Deaths/participants | HR (95% CI) | P value | P for interaction |
|---|---|---|---|---|---|
| **Overall** | | 202/978 vs 376/3475 | 1.31 (1.09–1.57) | 0.003 | |
| **Sex** | | | | | 0.030 |
| | Female | 72/398 vs 138/1438 | 1.73 (1.31–2.29) | <0.001 | |
| | Male | 130/580 vs 238/2037 | 1.10 (0.85–1.42) | 0.479 | |
| **Age** | | | | | 0.037 |
| | <65 years | 71/583 vs 131/2533 | 2.03 (1.34–3.09) | <0.001 | |
| | ≥65 years | 131/395 vs 245/942 | 1.14 (0.89–1.45) | 0.302 | |
| **Race/ethnicity** | | | | | 0.056 |
| | Mexican American | 35/208 vs 25/542 | 3.23 (1.77–5.90) | <0.001 | |
| | Non-Hispanic Black | 58/284 vs 79/838 | 1.44 (0.93–2.22) | 0.103 | |
| | Non-Hispanic White | 86/284 vs 240/1376 | 1.22 (0.95–1.58) | 0.123 | |
| | Other Hispanic | 15/95 vs 17/317 | 1.45 (0.46–4.52) | 0.525 | |
| | Other race | 8/107 vs 15/402 | 0.54 (0.14–2.04) | 0.368 | |
| **Smoking status** | | | | | 0.210 |
| | Ever smoker | 121/512 vs 227/1912 | 1.45 (1.15–1.84) | 0.002 | |
| | Never smoker | 81/466 vs 149/1563 | 1.11 (0.80–1.53) | 0.536 | |
| **BMI** | | | | | 0.251 |
| | <25 kg/m² | 34/130 vs 161/1035 | 1.15 (0.80–1.67) | 0.452 | |
| | 25–<30 kg/m² | 71/293 vs 109/1249 | 1.79 (1.23–2.59) | 0.002 | |
| | ≥30 kg/m² | 97/555 vs 106/1191 | 1.11 (0.72–1.71) | 0.648 | |

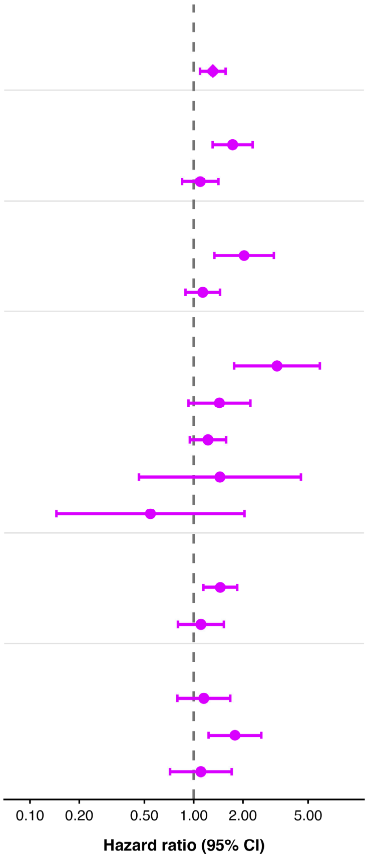


**Supplementary Figure S1.** Subgroup analyses of all-cause mortality comparing DM + P with P. Points represent HRs and horizontal bars represent 95% CIs estimated using fully adjusted survey-weighted Cox proportional hazards models. Deaths/participants are presented as DM + P vs P. The vertical dashed line indicates an HR of 1. P for interaction was obtained from the interaction term between DM–periodontitis status and each subgroup variable. HR, hazard ratio; CI, confidence interval; BMI, body mass index.

**DM + P vs DM**

| Subgroup | Category | Deaths/participants | HR (95% CI) | P value | P for interaction |
|---|---|---|---|---|---|
| **Overall** | | 202/978 vs 180/897 | 1.00 (0.73–1.35) | 0.984 | |
| **Sex** | | | | | 0.298 |
| | Female | 72/398 vs 84/504 | 1.19 (0.79–1.81) | 0.403 | |
| | Male | 130/580 vs 96/393 | 0.90 (0.62–1.32) | 0.587 | |
| **Age** | | | | | 0.629 |
| | <65 years | 71/583 vs 46/515 | 1.05 (0.67–1.66) | 0.826 | |
| | ≥65 years | 131/395 vs 134/382 | 0.91 (0.62–1.34) | 0.638 | |
| **Race/ethnicity** | | | | | 0.069 |
| | Mexican American | 35/208 vs 7/87 | 2.48 (1.20–5.15) | 0.014 | |
| | Non-Hispanic Black | 58/284 vs 43/222 | 1.23 (0.81–1.87) | 0.327 | |
| | Non-Hispanic White | 86/284 vs 107/397 | 0.95 (0.65–1.40) | 0.809 | |
| | Other Hispanic | 15/95 vs 10/97 | 1.18 (0.42–3.32) | 0.755 | |
| | Other race | 8/107 vs 13/94 | 0.22 (0.07–0.76) | 0.016 | |
| **Smoking status** | | | | | 0.466 |
| | Ever smoker | 121/512 vs 114/435 | 1.08 (0.78–1.48) | 0.649 | |
| | Never smoker | 81/466 vs 66/462 | 0.87 (0.51–1.49) | 0.607 | |
| **BMI** | | | | | 0.250 |
| | <25 kg/m² | 34/130 vs 38/117 | 0.70 (0.40–1.23) | 0.217 | |
| | 25–<30 kg/m² | 71/293 vs 51/234 | 1.34 (0.81–2.24) | 0.256 | |
| | ≥30 kg/m² | 97/555 vs 91/546 | 0.92 (0.61–1.39) | 0.704 | |

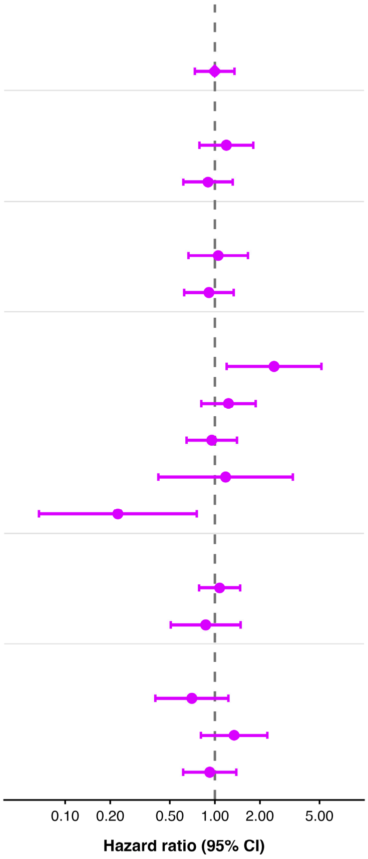


**Supplementary Figure S2.** Subgroup analyses of all-cause mortality comparing DM + P with DM. Points represent HRs and horizontal bars represent 95% CIs estimated using fully adjusted survey-weighted Cox proportional hazards models. Deaths/participants are presented as DM + P vs DM. The vertical dashed line indicates an HR of 1. P for interaction was obtained from the interaction term between DM–periodontitis status and each subgroup variable. HR, hazard ratio; CI, confidence interval; BMI, body mass index.

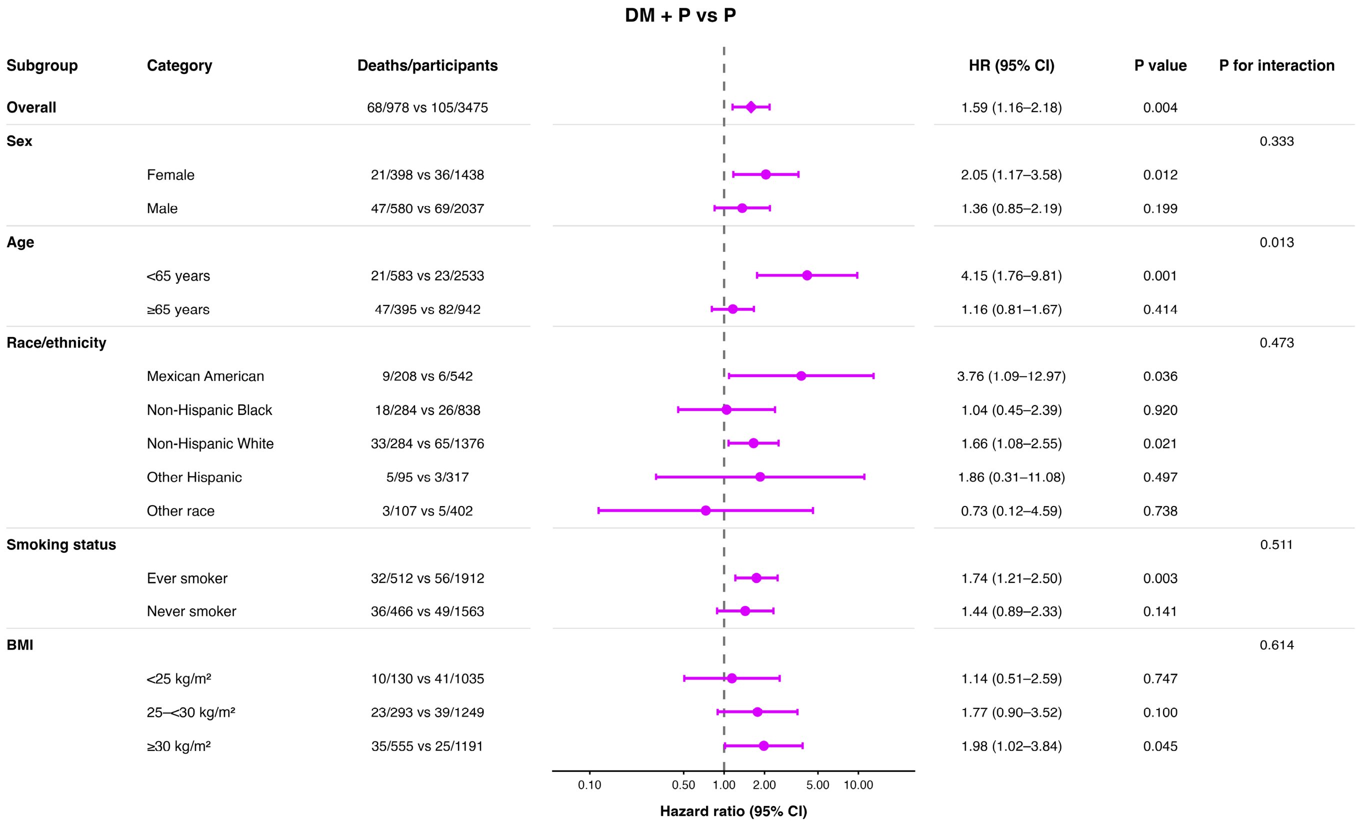


**Supplementary Figure S3.** Subgroup analyses of cardiovascular mortality comparing DM + P with P. Points represent cause-specific HRs and horizontal bars represent 95% CIs estimated using fully adjusted survey-weighted Cox proportional hazards models, with noncardiovascular deaths censored. Deaths/participants are presented as DM + P vs P. The vertical dashed line indicates an HR of 1. P for interaction was obtained from the interaction term between DM–periodontitis status and each subgroup variable. HR, hazard ratio; CI, confidence interval; BMI, body mass index.

**DM + P vs DM**

| Subgroup | Category | Deaths/participants | HR (95% CI) | P value | P for interaction |
|---|---|---|---|---|---|
| **Overall** | | 68/978 vs 61/897 | 1.08 (0.67–1.76) | 0.741 | |
| **Sex** | | | | | 0.655 |
| | Female | 21/398 vs 29/504 | 1.22 (0.60–2.44) | 0.584 | |
| | Male | 47/580 vs 32/393 | 1.04 (0.64–1.68) | 0.878 | |
| **Age** | | | | | 0.911 |
| | <65 years | 21/583 vs 14/515 | 1.07 (0.32–3.59) | 0.911 | |
| | ≥65 years | 47/395 vs 47/382 | 0.99 (0.60–1.65) | 0.970 | |
| **Race/ethnicity** | | | | | 0.223 |
| | Mexican American | 9/208 vs 2/87 | 3.08 (0.70–13.64) | 0.138 | |
| | Non-Hispanic Black | 18/284 vs 13/222 | 1.57 (0.67–3.70) | 0.302 | |
| | Non-Hispanic White | 33/284 vs 39/397 | 1.08 (0.58–2.04) | 0.802 | |
| | Other Hispanic | 5/95 vs 1/97 | 3.20 (0.35–29.50) | 0.304 | |
| | Other race | 3/107 vs 6/94 | 0.20 (0.04–1.06) | 0.059 | |
| **Smoking status** | | | | | 0.512 |
| | Ever smoker | 32/512 vs 39/435 | 0.99 (0.59–1.64) | 0.954 | |
| | Never smoker | 36/466 vs 22/462 | 1.29 (0.60–2.78) | 0.514 | |
| **BMI** | | | | | 0.587 |
| | <25 kg/m² | 10/130 vs 13/117 | 0.68 (0.26–1.80) | 0.443 | |
| | 25–<30 kg/m² | 23/293 vs 16/234 | 1.32 (0.58–2.99) | 0.508 | |
| | ≥30 kg/m² | 35/555 vs 32/546 | 1.10 (0.56–2.15) | 0.774 | |

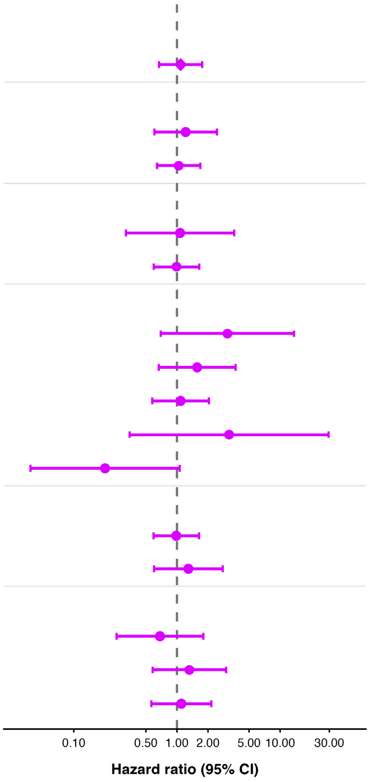


**Supplementary Figure S4.** Subgroup analyses of cardiovascular mortality comparing DM + P with DM. Points represent cause-specific HRs and horizontal bars represent 95% CIs estimated using fully adjusted survey-weighted Cox proportional hazards models, with noncardiovascular deaths censored. Deaths/participants are presented as DM + P vs DM. The vertical dashed line indicates an HR of 1. P for interaction was obtained from the interaction term between DM–periodontitis status and each subgroup variable. HR, hazard ratio; CI, confidence interval; BMI, body mass index.

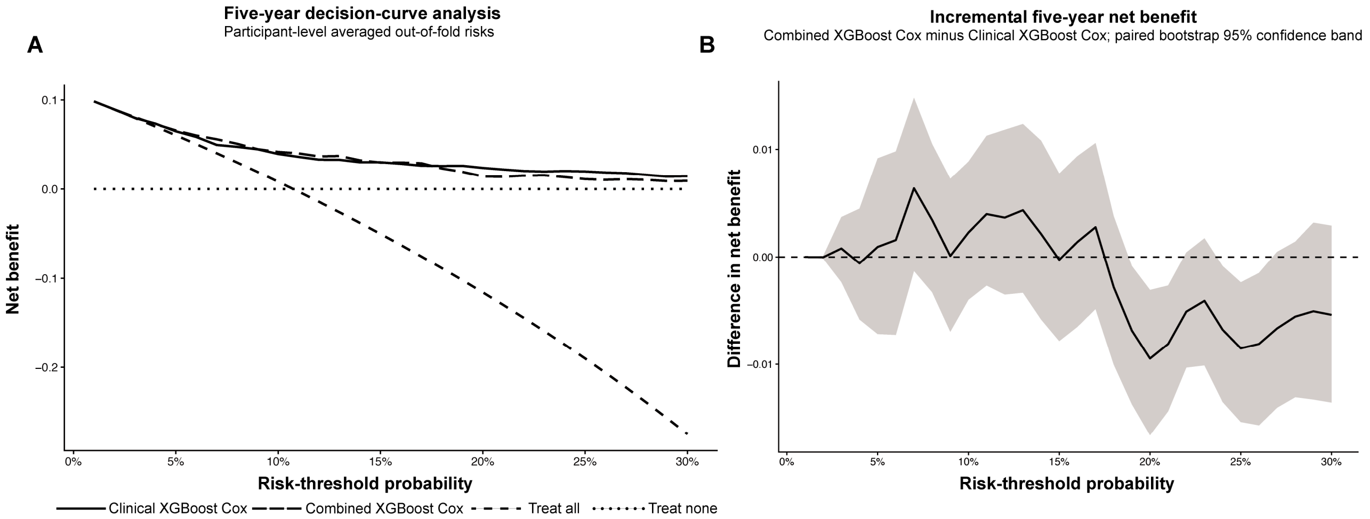


**Supplementary Figure S5.** Five-year decision-curve analysis comparing the Clinical and Combined XGBoost Cox models. (A) Net benefit based on participant-averaged out-of-fold 5-year mortality risks across threshold probabilities from 0.01 to 0.30. The solid line represents the Clinical XGBoost Cox model, the long-dashed line the Combined XGBoost Cox model, and the dashed and dotted lines the treat-all and treat-none strategies, respectively. (B) Combined-minus-Clinical difference in net benefit. The solid line represents the observed difference, and the shaded area represents the pointwise percentile 95% confidence interval obtained from paired participant-level bootstrap samples. Confidence intervals were not adjusted for multiplicity across risk thresholds.

# References


56. White, I.R.; Royston, P.; Wood, A.M. Multiple Imputation Using Chained Equations: Issues and Guidance for Practice. Stat. Med. 2011, 30, 377–399, doi:10.1002/sim.4067.

57. Marshall, A.; Altman, D.G.; Holder, R.L.; Royston, P. Combining Estimates of Interest in Prognostic Modelling Studies after Multiple Imputation: Current Practice and Guidelines. BMC Med. Res. Methodol. 2009, 9, 57, doi:10.1186/1471-2288-9-57.

58. Inker, L.A.; Eneanya, N.D.; Coresh, J.; Tighiouart, H.; Wang, D.; Sang, Y.; Crews, D.C.; Doria, A.; Estrella, M.M.; Froissart, M.; et al. New Creatinine- and Cystatin C-Based Equations to Estimate GFR without Race. N. Engl. J. Med. 2021, 385, 1737–1749, doi:10.1056/NEJMoa2102953.

59. Kidney Disease: Improving Global Outcomes (KDIGO) CKD Work Group KDIGO 2024 Clinical Practice Guideline for the Evaluation and Management of Chronic Kidney Disease. Kidney Int. 2024, 105, S117–S314, doi:10.1016/j.kint.2023.10.018.

60. Deng, R.; Zhu, S.; Fan, B.; Chen, X.; Lv, H.; Dai, Y. Exploring the Correlations between Six Serological Inflammatory Markers and Different Stages of Type 2 Diabetic Retinopathy. Sci. Rep. 2025, 15, 1567, doi:10.1038/s41598-025-85164-2.

61. Zinellu, A.; Collu, C.; Nasser, M.; Paliogiannis, P.; Mellino, S.; Zinellu, E.; Traclet, J.; Ahmad, K.; Mangoni, A.A.; Carru, C.; et al. The Aggregate Index of Systemic Inflammation (AISI): A Novel Prognostic Biomarker in Idiopathic Pulmonary Fibrosis. J. Clin. Med. 2021, 10, 4134, doi:10.3390/jcm10184134.

62. Chen, P.; Zhu, M.; Guo, M.; Shi, D.; Chen, Z.; Du, J. Platelet to High Density Lipoprotein Cholesterol Ratio Is Associated with Diabetes and Prediabetes in NHANES 2005 to 2018. Sci. Rep. 2024, 14, 30082, doi:10.1038/s41598-024-81637-y.

63. Onodera, T.; Goseki, N.; Kosaki, G. [prognostic nutritional index in gastrointestinal surgery of malnourished cancer patients]. Nihon Geka Gakkai Zasshi 1984, 85, 1001–1005.
